# A group of novel VEGF splice variants as alternative therapeutic targets in renal cell carcinoma

**DOI:** 10.1002/1878-0261.13401

**Published:** 2023-04-18

**Authors:** Christopher Montemagno, Jérôme Durivault, Cécile Gastaldi, Maeva Dufies, Valérie Vial, Xingkang He, Damien Ambrosetti, Anna Kamenskaya, Sylvie Negrier, Jean‐Christophe Bernhard, Delphine Borchiellini, Yihai Cao, Gilles Pagès

**Affiliations:** ^1^ Biomedical Department Centre Scientifique de Monaco Monaco; ^2^ University Côte d'Azur, Institute for Research on Cancer and Aging of Nice (IRCAN), CNRS UMR 7284; INSERM U1081, Centre Antoine Lacassagne Nice France; ^3^ LIA BAHN, CSM‐UVSQ Monaco Monaco; ^4^ Department of Microbiology, Tumor and Cell Biology Karolinska Institute Stockholm Sweden; ^5^ Department of Pathology University Côte d'Azur, CHU Nice Nice France; ^6^ Kekkan Biologics Strasbourg France; ^7^ Centre Léon Bérard University Claude Bernard Lyon Lyon France; ^8^ Department of Urology Centre Hospitalier Universitaire (CHU) de Bordeaux France; ^9^ Department of Medical Oncology Centre Antoine Lacassagne Nice France; ^10^ LIA ROPSE, Laboratoire International Associé Université Côte d'Azur—Centre Scientifique de Monaco Nice France

**Keywords:** alternative splicing, angio/lymphangiogenesis, renal cell carcinoma, resistance to antiangiogenics, VEGF

## Abstract

The efficacy of anti‐angiogenic treatment by targeting VEGF/VEGF receptors in metastatic clear cell renal cell carcinoma (ccRCC) varies from patient to patient. Discovering the reasons behind this variability could lead to the identification of relevant therapeutic targets. Thus, we investigated the novel splice variants of VEGF that are less efficiently inhibited by anti‐VEGF/VEGFR targeting than the conventional isoforms. By *in silico* analysis, we identified a novel splice acceptor in the last intron of the *VEGF* gene resulting in an insertion of 23 bp in VEGF mRNA. Such an insertion can shift the open‐reading frame in previously described splice variants of VEGF (VEGF_XXX_), leading to a change in the C‐terminal part of the VEGF protein. Next, we analysed the expression of these alternatively spliced VEGF new isoforms (VEGF_XXX/NF_) in normal tissues and in RCC cell lines by qPCR and ELISA, and we investigated the role of VEGF_222/NF_ (equivalent to VEGF_165_) in physiological and pathological angiogenesis. Our *in vitro* data demonstrated that recombinant VEGF_222/NF_ stimulated endothelial cell proliferation and vascular permeability by activating VEGFR2. In addition, VEGF_222/NF_ overexpression enhanced proliferation and metastatic properties of RCC cells, whereas downregulation of VEGF_222/NF_ resulted in cell death. We also generated an *in vivo* model of RCC by implanting RCC cells overexpressing VEGF_222/NF_ in mice, which we treated with polyclonal anti‐VEGF_XXX/NF_ antibodies. VEGF_222/NF_ overexpression enhanced tumour formation with aggressive properties and a fully functional vasculature, while treatment with anti‐VEGF_XXX/NF_ antibodies slowed tumour growth by inhibiting tumour cell proliferation and angiogenesis. In a patient cohort from the NCT00943839 clinical trial, we investigated the relationship between plasmatic VEGF_XXX/NF_ levels, resistance to anti‐VEGFR therapy and survival. High plasmatic VEGF_XXX/NF_ levels correlated with shorter survival and lower efficacy of anti‐angiogenic drugs. Our data confirmed the existence of new VEGF isoforms that could serve as novel therapeutic targets in patients with RCC that are resistant to anti‐VEGFR therapy.

## Graphical abstract

We identified new splice variants of VEGF (VEGF_XXX/NF_) by bioinformatic analysis and molecular approaches, and we observed that VEGF_XXX/NF_ stimulated angio/lymphangiogenesis and vascular permeability. High levels of VEGF_XXX/NF_ correlated with shorter survival and resistance to anti‐angiogenic drugs (AAD) in patients with metastatic renal cancer. Thus, VEGF_XXX/NF_ could serve as a relevant therapeutic target, a prognostic marker and a predictive marker for the efficacy of AAD.

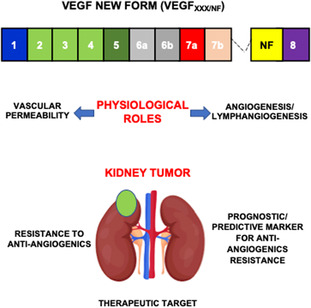

**Abbreviations**
ANOVA: analysis of variance
ATP: adenosine triphosphate
BSA: bovine serum albumin
ccRCC: clear cell renal cell carcinoma
DNA: deoxyribonucleic acid
EC: endothelial cell
ELISA: enzyme‐linked immunosorbent assay
Flt‐1: FMS‐like tyrosine kinase 1
HIF: hypoxia‐inducible factor
HRP: horseradish peroxidase
KDR: kinase insert domain receptor
Lyve I: lymphatic vessel endothelial receptor 1
mccRCC: metastatic clear cell renal cell carcinoma
MET: c‐MET tyrosine‐kinase receptor
mRNA: messenger ribonucleic acid
NRP: neuropilin
OS: overall survival
PBS: phosphate‐buffered saline
PFS: progression‐free survival
qPCR: quantitative polymerase chain reaction
shRNA: short‐hairpin ribonucleic acid
TIME: telomerase‐immortalized microvascular endothelial cells
TKI: tyrosine kinase inhibitor
VEGF: vascular endothelial growth factor
VEGF_222/NF_
: specific VEGF_XXX/NF_ isoform derived from VEGF_165_ (recombinant or from an expression vector)
VEGFR: vascular endothelial growth factor receptor
VEGF_XXX/NF_
: generic term to describe all VEGF_XXX/NF_ isoform from the already described spliced forms
VHL: von‐Hippel Lindau
αSMA: α smooth muscle actin

## 1 Background

Tumours require a continuous supply of nutrients and oxygen as well as the ability to excrete carbon dioxide and waste products. These needs are met by the tumour‐associated neo‐vasculature through angiogenesis [1]. Angiogenesis is temporarily activated during physiological processes such as the female reproductive cycle or wound healing [2]. During tumour progression, angiogenesis creates a vascular network that allows tumour cells to spread [3]. In angiogenesis, there is a balance between pro‐ and anti‐angiogenic factors. In cancer, the balance is shifted in favour of pro‐angiogenic factors resulting in abnormal neovascularization. The discovery of the vascular endothelial growth factor (VEGF), as one of the most important pro‐angiogenic factors, in 1989 marked a breakthrough in the understanding of the mechanisms of angiogenesis [4, 5, 6, 7]. VEGF stimulates angiogenesis and vascular permeability through the activation of two tyrosine‐kinase receptors, VEGFR1/Flt1 and VEGFR2/KDR [8].

Most clear cell renal cell carcinomas (ccRCC) exhibit inactivation of the von Hippel–Lindau gene (VHL), leading to stabilization of hypoxia‐inducible factors 1 and 2 alpha (HI1/2α) and consequent overexpression of VEGF [9, 10]. However, ccRCC with the wild‐type form of VHL appear more aggressive [11]. The VEGF/VEGFRs signalling pathway is a key mediator of clear ccRCC aggressiveness [12]. The therapeutic management of ccRCC patients depends on the stage of the disease. In non‐metastatic patients, surgery is the standard treatment and adjuvant therapy is only relevant for patients with local invasion [13]. In the metastatic phase, ccRCC is unfortunately refractory to conventional chemo/radiotherapy [14]. However, hypervascularization favours the use of anti‐angiogenic therapies targeting VEGF or its receptors. In 2007, several clinical trials demonstrated their efficacy with an improvement in progression‐free survival (PFS) compared to interferon alpha, the reference treatment at that time. Following the completion of clinical trials, the Food and Drug Administration (FDA) approved the small ATP mimetics sorafenib [15] and sunitinib [16] for the treatment of metastatic ccRCC. In recent years, many other tyrosine kinase inhibitors (TKIs) have been approved for the treatment of metastatic ccRCC, including pazopanib [17], tivozanib [18], cabozantinib [19] and lenvatinib [20]. The FDA has also approved bevacizumab, an anti‐VEGF monoclonal antibody, for the treatment of metastatic ccRCC in combination with interferon alpha [21]. Given the important role played by tumour neovascularization, bevacizumab has also been approved for the treatment colorectal cancer [22], non‐small‐cell lung cancer [23], breast cancer [24] and ovarian cancer [25] in combination with standard chemotherapy. Although bevacizumab/chemotherapy‐dependent increased PFS, its limited effect on overall survival (OS) led the FDA to lose approval for breast cancer [26]. In renal cell carcinoma, bevacizumab in combination with interferon lost its FDA approval in 2016, but was recently approved in combination with the anti‐PD‐L1 antibody atezolizumab [27]. ccRCC has also become a paradigm for combining immunotherapies [28] or combinations of immunotherapies with anti‐angiogenics. However, biomarkers are needed to stratify patients who might benefit from these different therapies. Molecular subgroups have also been defined for their sensitivity to TKIs or immunotherapies [29, 30, 31]. These molecular subgroups may be relevant for repositioning specific therapies [32]. The complexity of VEGF biology may partly explain the limited efficacy of bevacizumab compared to multi‐target tyrosine kinase inhibitors. VEGF is regulated during all processes of its expression, including transcription of its gene, splicing of its pre‐mRNA, stabilization/destabilization of its mRNA and translation [33]. Alternative splicing of VEGF pre‐mRNA results in mRNAs encoding for pro‐angiogenic isoforms known as VEGF_XXX_ (VEGF_121_, VEGF_165_, VEGF_189_ and VEGF_206_, where XXX corresponds to the number of amino acids minus the signal peptide of each isoform). In 2002, an alternative splice site was discovered in exon 8 of the human VEGF gene, resulting in the VEGF_XXXb_ family. VEGF_XXXb_ isoforms that differ from VEGF_XXX_ in the last six amino acids (CDKPRR for VEGF_XXX_, SLTRKD for VEGF_XXXb_) [34]. While VEGF_XXX_ isoforms have pro‐angiogenic properties, VEGF_XXXb_ isoforms were considered anti‐angiogenic. The same controversy has been described for VEGF‐Ax, which results from a translational readthrough the stop codon and generates a longer VEGF isoform [35, 36].

In the current study, we describe for the first time the existence of an alternative splice acceptor site in the seventh intron. The resulting alternative splicing leads to the production of at least four new isoforms of VEGF, VEGF_XXX/NF_. The predominant isoform, VEGF_222/NF_, exhibits physiological pro‐angiogenic and pro‐lymphangiogenic properties. Its expression was detected in several ccRCC cell lines. It stimulated tumour growth and metastatic dissemination through the development of networks of mature blood and lymphatic vessels. In a model of experimental ccRCC resistant to bevacizumab, inhibition of VEGF_XXX/NF_ with specific polyclonal antibodies delayed tumour growth. In addition, VEGF_XXX/NF_ expression predicted poor prognosis and resistance to anti‐angiogenic drugs in metastatic ccRCC patients.

Since the discovery of VEGF in 1989, this new type of regulation has not been described. The existence of this biologically different isoform turns the VEGF field on its head. So, the secrets of VEGF are still not revealed 30 years after its discovery.

## 2 Materials and methods

### 2.1 RT‐PCR and RT‐qPCR

**Fig. 1:**
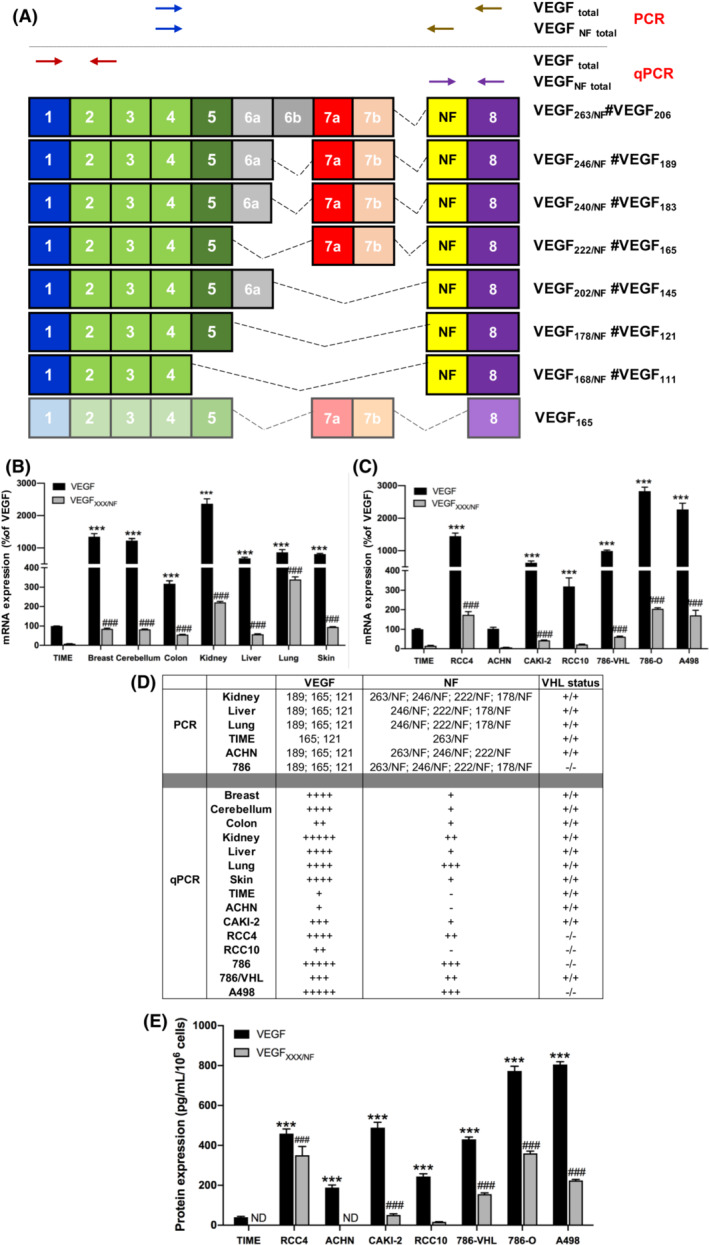
VEGF_XXX/NF_ isoforms are expressed in normal tissues and cancer cells. (A) VEGF_XXX/NF_ splice variants. Primers used for RT‐(q)PCR analyses are indicated by the arrows. The names of the different VEGF_XXX_ isoforms and the corresponding VEGF_XXX/NF_ isoforms are indicated. (B) RT‐qPCR analyses of the expression of the different VEGF and VEGF_XXX/NF_ in normal tissues; ***P < 0.001 vs VEGF in TIME cells; ^###^ *P* < 0.001 vs VEGF_XXX/NF_ in TIME cells. (C) RT‐qPCR analyses of VEGF and VEGF_XXX/NF_ expression in RCC cell lines (RCC4, ACHN, CAKI‐2, RCC10, 786‐VHL, 786‐O, A498). Results are expressed as percent (%) of VEGF in TIME cells. ***P < 0.001 vs VEGF in TIME cells; ^###^ *P* < 0.001 vs VEGF_XXX/NF_ in TIME cells. (D) Summary table of VEGF and VEGF_XXX/NF_ expression levels in normal tissue and RCC cells. (E) ELISA of VEGF (Peprotech) and VEGF_XXX/NF_ (rabbit anti‐VEGF_XXX/NF_ clone # 2) expression in supernatant of RCC cells ***P < 0.001 vs VEGF in TIME cells; ^###^ *P* < 0.001 vs VEGF_XXX/NF_ in RCC10 (two‐way ANOVA). Data are presented as the mean ± standard error of the mean (SEM). All experiments were performed with at least three biological duplicates (*n* = 3) for each group in triplicate.

RT‐PCR and RT‐qPCR analyses were performed with mRNA from human tissue (Biochain^®^; BioChain Institute Inc., Newark, CA, USA) and cell lines. The mRNAs were prepared using a Nucleospin RNA kit (Macherey‐Nagel, Düren, Germany), and cDNA synthesis was performed using a Maxima First Strand cDNA Synthesis Kit for RT‐qPCR, with dsDNase (Fischer Scientific, Waltham, MA, USA). PCR analysis was performed on a Biometra thermal cycler with PrimeSTAR^®^ GXL DNA polymerase (Takara Bio, Shiga, Japan). Quantitative PCR analysis was performed on an Applied Biosystems StepOnePlus™ system with the reagents TB Green^®^ premix Ex Taq™ (Tli RNase H Plus; Takara Bio). The primers used are listed in Online Resource 12. All amplicons shown in Fig. 1 were verified by sequencing (Eurofins genomics, Ebersberg). The expression values were determined using the Δ*C*_t_ method and normalized to the reference gene 36B4. The results of the RT‐qPCR analyses were expressed as percent of total VEGF. The following oligonucleotides were used in PCR/qPCR experiments.

**Table:** 

	Forward	Reverse	Size (bp)
qPCR			
hVEGF_XXX/NF Total_	CCTTTGTTTTCCATTTCCCT	TCTGTCGATGGTGATGGTGTG	169
hVEGF_Total_	TTTCTGCTGTCTTGGGTGCATTGG	ACCACTTCGTGATGATTCTGCCCT	116
hVEGF_165_	TGTTTGTACAAGATCCGCAGACGTG	CTCGGCTTGTCACATCTGCAAGTACG	112
hVEGF_121_	ATCTTCAAGCCATCCTGTGTGC	TCGGCTTGTCACATTTTTCTTG	223
hVEGF_168/NF_	CCCACTGAGGAGTCCAACATCAC	GGAAAACAAAGGCTGCATTCACATTTG	119
hVEGF_178/NF_	GCGGATCAAACCTCACCAAGG	GGAAAACAAAGGTTTTCTTGTCTTGCTC	114
hVEGF_202/NF_	GCGGATCAAACCTCACCAAGG	GGAAAACAAAGGACGCTCCAGGACTTATAC	186
hVEGF_222/NF_	CAAGACAAGAAAATCCCTGTGGGC	GGAAATGGAAAACAAAGGCTGCAAGTAC	163
hVEGF_240/NF_	CAAGAAATCCCGTCCCTGTGGG	GGAAATGGAAAACAAAGGCTGCAAGTAC	162
hVEGF_246/NF_	CCTGGAGCGTTCCCTGTGG	GGAAATGGAAAACAAAGGCTGCAAGTAC	160
hVEGF_263/NF_	CCTGGAGCGTGTACGTTGGTG	GGAAATGGAAAACAAAGGCTGCAAGTAC	211
h36b4	CAGATTGGCTACCCAACTGTT	GGCCAGGACTCGTTTGTACC	69
m36b4	AGATTCGGGATATGCTGTTGGC	TCGGGTCCTAGACCAGTGTTC	109
mCD31	ACGCTGGTGCTCTATGCAAG	TCAGTTGCTGCCCATTCATCA	109
mVEGFR2	TTTGGCAAATACAACCCTTCAGA	GCAGAAGATACTGTCACCACC	133
mαSMA	GTCCCAGACATCAGGGAGTAA	TCGGATACTTCAGCGTCAGGA	102
mVEGFR3	CGAGTCGGAGCCTTCTGAGG	GCAGTCCAGCAATAGGGGGT	151
mLYVE‐1	CAG CACACTAGCCTGGTGTTA	CGCCCATGATTCTGCATGTAGA	112
mPROX1	TGCGTGTTGCACCACAGAATA	AGAAGGGTTGACATTGGAGTGA	138
PCR			
hVEGF_XXX/NF Total_	GCGGATCAAACCTCACCAAGG	GCTTGTCACATCTGAGGGAAATG	391/340/322/268/208/136/106
hVEGF_Total_	GCGGATCAAACCTCACCAAGG	TCTGTCGATGGTGATGGTGTG	526/475/457/403/343/271/241 503/452/434/380/320/248/218
h36B4	CAGATTGGCTACCCAACTGTT	GGCCAGGACTCGTTTGTACC	69

### 2.2 Protein production and production of anti‐VEGF_XXX_ _/NF_ antibodies

Recombinant VEGF_222/NF_ was produced in HEK293 cells from ProteoGenix (Schiltigheim, France). The native cDNA and protein sequences and the recombinant optimized ones are shown in Fig. S1A–D.

Anti‐VEGF_NF_ polyclonal antibodies (anti‐VEGF_NF#1 and anti‐VEGF_NF#2) were produced in rabbits by ProteoGenix. The following two peptides were used for immunization of rabbits: DVTSRGGEPGRRKE and SGFREPDLSPGKTD. The same peptides were used to immunize Balb/C mice.

### 2.3 Plasmids

To generate lentiviral VEGF_222/NF_ expression plasmids, the VEGF_222/NF_ gene sequence was subcloned into pLenti6.3/TO/V5‐GW/LacZ‐Blasti (ThermoFischer, A11144; Fig. S1A,B). To generate lentiviral shRNA plasmids, the pLKO.1‐TRC cloning vector was used, a kind gift from Dr D. Roots (Addgene plasmid, [37]). The following shRNA sequences were used: shScramble: 5′‐CCTAAGGTTAAGTCGCCCTCG‐3′; shVEGFNF#1: 5′‐GCCTTTGTTTTCCATTTCC‐3′; shVEGFNF#2: 5′‐CATTTCCCTCAGATGTGACAA‐3′; shNRP1 5′‐TGTGGATGACATTAGTATTAA‐3′; shNRP2 5′‐CCTCAACTTCAACCCTCACTT‐3′. The shScramble was a gift from D. Sabatini (Addgene plasmid, [38]). All plasmids were verified by sequencing (Eurofins Genomics).

### 2.4 Lentiviral production and transduction

Lentiviruses were obtained by triple transfection of HEK‐293T cells with a lentiviral transfer vector (pLenti6.3/TO/V5‐GW/LacZ (ThermoFischer Scientific, A11144) for overexpression experiments and pLKO.1 for shRNA experiments), and the packaging plasmids psPAX2 (Addgene plasmid #12260) and pMD2.G (Addgene plasmid #12259) were a kind gift from Didier Trono, in a ratio of 0.3 : 0.3 : 0.1. Transfection was performed using jetPEI^®^ reagent. The viral supernatant was collected 48 h after transfection, filtered through a 0.22 μm philtre and added to the target cells.

### 2.5 Cell lines

Renal cell carcinoma cell lines ACHN (RRID: CVCL_1067), A498 (RRID: CVCL_1056), CAKI‐2 (RRID: CVCL_0235), RCC4 (RRID: CVCL_0498), 786‐O (RRID: CVCL_1051), RENCA (RRID: CVCL_2174), Telomerase‐immortalized microvascular endothelial cells (TIME, RRID: CVCL_0047), DAOY (medulloblastoma, RRID: CVCL_1167), MDA‐MB231 (breast, RRID: CVCL_0062) and MiaPaca‐2 (pancreas, RRID: CVCL_0428) tumour cells were from ATCC^®^. RCC10 cells were a kind gift from Pr W.H. Kaelin (Dana‐Farber Cancer Institute, Boston, MA, USA). 786‐O expressing VHL were a gift from Dr N. Mazure [39]. TIME cells were cultured in vascular cell basal medium (ATCC^®^ PCS‐100‐030™) supplemented with microvascular endothelial cell growth kit (ATCC^®^ PCS‐100‐041™). Primary human dermal lymphatic endothelial cells (HDLECs) were from Promocell. All cell lines were regularly tested for mycoplasma. Regular thawing of the originally authenticated ATCC cell lines and analysis of the expression of specific genes are part of the authentication process.

### 2.6 Cell proliferation assays

TIME cells (50 000) were seeded in 6‐well plates in Endothelial Cell Growth Medium (Promocell) containing 0.5% FBS. Twenty‐four hours later, cells were treated with VEGF_222/NF_ (100 ng·mL^−1^) (day 0) and were counted at days 0, 1, 3, 5 and 7. For ACHN proliferation, ACHN‐LacZ and ACHN‐VEGF_222/NF_ cells (20,000) were seeded in 6‐well plates in DMEM medium containing 0.5% FBS. Cells were counted at days 0, 1, 3, 5 and 7.

### 2.7 Clonogenicity

ACHN and 786‐O cells were washed twice with PBS and reseeded at a density of 8000 (ACHN) or 4000 (786‐O) cells/well in 6‐well plates. Twenty‐four hours later, cells were transduced with shVEGF_NF_ (#1 and #2). Twenty‐four hours later, the media were changed. After 7 days, colonies were stained with 0.1% crystal violet. The plates were photographed.

### 2.8 Migration assay

The confluent TIME cells were starved for 2 h. Then, the cell monolayer was disrupted to create a scratch wound and rinsed with PBS before being treated with VEGF_222/NF_ (100 ng·mL^−1^). Images were acquired immediately after scratching (0 h) using phase contrast microscopy (Evos™ xl core, ThermoFischer) and after 3, 6, 9 and 12 h. The images were analysed with the Java‐based program imagej34 (Bethesda, MD, USA), and the distance was measured after 0, 3, 6, 9 and 12 h.

### 2.9 Tests of endothelial permeability

*In vitro* permeability was studied as described [40]. Briefly, TIME cells were grown on membranes of 6.5 mm Transwell inserts (0.4 μm pore size, Corning^®^). Confluent cells were starved for 2 h and then treated with VEGF_222/NF_ (100 ng·mL^−1^) for 20 min in the upper chamber. The medium was then aspirated and replaced with streptavidin‐HRP‐containing medium (1/200, R&D Systems, Minneapolis, MN, USA) for 5 min. The inserts were removed, and 20 μL of medium from the lower chamber was transferred to a 96‐well plate. TMB substrate (50 μL, Sigma, Saint‐Quentin‐Fallavier Cedex, France) was added for 5 min, and the reaction was stopped with sulphuric acid. Absorbance at 450 nm was determined using an ELISA reader (ThermoScientific, Multiskan FC).

### 2.10 Immunoblotting

Cells were lysed in Laemmli buffer containing 2% SDS, 10% glycerol, 60 mm Tris–HCl (pH 6.8) and 1× Halt™ phosphatase inhibitor cocktail (Thermo Fischer). DNA was fragmented by sonication. Lysates supplemented with 0.002% bromophenol blue and 100 mm DTT were heated to 96 °C, separated by SDS/PAGE and transferred to PVDF membranes (Millipore, Saint‐Quentin‐Fallavier Cedex, France). Membranes were tested with the following antibodies: Akt, 9272S, CST; p‐Akt Ser473, CST, 4051S; ‐Akt Thr308 CST, 5106S ARD1, Santa Cruz Biotechnology, Santa Cruz, CA, USA, sc‐373920; HSP90, Pierce, Saint‐Quentin‐Fallavier Cedex, France, MA1‐10373; p44/42 MAPK (pERK1/2), CST, 9102S; ERK1/2, Sigma Aldrich, M8159; pVEGFR2 (Tyr1175), CST, 2478S; VEGFR2, CST, 2479S. Reactive bands were visualized with chemiluminescent Western Immobilon™ HRP substrate (Merck Millipore^®^).

### 2.11 Immunofluorescence

Tumour sections (5‐μm cryostat sections) were fixed in 4% paraformaldehyde for 10 min at room temperature and blocked in 1% horse serum in Tris‐buffered saline (TBS) for 1 h. The sections were then incubated overnight at 4 °C with anti‐rabbit LYVE‐1 polyclonal antibody (Ab14917, 1 : 200; Abcam, Cambridge, MA, USA) or mouse CD31 monoclonal antibody (clone MEC 13.3, 1 : 1000; BD Pharmingen, San Diego, CA, USA) and mouse anti‐α‐smooth muscle actin monoclonal antibody (αSMA A2547, 1 : 1000; Sigma) or anti‐Ki67 (ab16667, 1 : 500; Abcam). Preparations were mounted and analysed using a Leica microscope (Leica DMI4000B; Leica, Richmond, IL, USA) and counted at 10× magnification (CD31/αSMA) and 40× magnification (Ki67). The results are given as the number of vessels per mm^2^ of sections.

### 2.12 ELISA

Saturation binding experiments were performed on 96‐well plates coated with human VEGFR1, VEGFR2, VEGFR3, NRP1 and NRP2 (100 ng per well: R&D Systems). Serial dilutions of VEGF_222/NF_ were incubated at room temperature for 1 h (RT) before washing three times with PBS‐Tween 0.05%. Anti‐VEGF_NF_ (0.25 μg·mL^−1^, rabbit) was then incubated for 1 h at RT, followed by anti‐rabbit HRP conjugate. The ELISA was displayed after addition of TMB. VEGF_222/NF_ binding curves were fitted using a nonlinear regression equation (specific binding: *y* = *B*_max_*x/(*K*_D_ + *x*)), where x is the ligand concentration, *K*_D_ is the dissociation constant and *B*_max_ is the maximum number of binding sites (graphpad prism version 8, software, Boston, MA, USA) to determine *K*_D_ values.

For RCC cell lines, total VEGF and total VEGF_XXX/NF_ levels were measured by ELISA. RCC cell lines were seeded in 6‐well plates (500 000) and grown in DMEM medium containing 0.5% FBS for 48 h. VEGF assays were performed using the human VEGF standard development kit TMB ELISA (Peprotech^®^, Cranbury, NJ, USA; Human VEGF_165_ Standard TMB ELISA Development Kit, 900‐T10) according to the manufacturer's recommendations. For VEGF_XXX/NF_, rabbit anti‐VEGF_XXX/NF_ antibody (clone #2, 1.5 μg·mL^−1^) was applied to the plastic of 96‐well plates overnight at 4 °C in PBS. Samples were incubated in PBS‐0.5% BSA‐0.05% Tween for 1 hour at room temperature. Detection was performed as for the VEGF assay (Peprotech^®^, 900‐T10). The ELISA was performed to determine the affinity of bevacizumab. Binding at saturation was determined on 96‐well plates coated with recombinant VEGF_165_ and VEGF_222/NF_ protein (100 ng per well). Serial dilutions of bevacizumab (Roche^®^, Vaud, Switzerland) were incubated for 2 h at room temperature before washing five times with PBS‐Tween 0.05%. The ELISA was detected after addition of TMB followed by incubation with an anti‐human HRP.

### 2.13 Octet experiments

Octet was performed by the Deeptope company (Paris, France). The Octet is an instrument capable of measuring an interaction between two partners. In the case of a receptor/ligand interaction, one of the two partners is immobilized on a sensor that has a functionalized surface. The sensor is immersed in a solution containing the second partner at a given concentration. Association and dissociation signals are recorded and analysed to extract association and dissociation constants as well as the KD. For such experiments, receptors/coreceptors need to contain a way to favour interaction with a substrate. The different receptors/co‐receptors were then tagged with biotin except for NRP2 which was tagged with an Fc fragment.

Materials and buffers used:

- Instrument used: Octet^®^ RED96
- Sensors used: Anti‐human Fc (AHC) biosensors and streptavidin (SA) biosensors
- Buffer used (Kinetic Buffer = KB): PBS + BSA 0.1% + Tween 20 0.02%
- Sequence used: Specified in each individual experiment design
- Analytical software used: dataanalysisht v10 (FortéBio, Dallas, TX, USA)

Biotinylated VEGFR1, VEGFR2, VEGFR3 and NRP1 were from Acrobio (Newark, DE, USA). Fc‐tagged NRP2 was from Novus Bioscience (Centennial, CO, USA).

Sensors coated with anti‐hFc (NRP2) or streptavidin VEGFR1, VEGFR2, VEGFR3 and NRP1 are used. The proteins are first loaded onto the sensors, then after a baseline step in the Kinetic Buffer (KB), the sensors are dipped into wells containing varying concentrations of VEGF‐NF1, VEGF‐A or VEGF‐C. Then, the dissociation is measured by dipping the sensors into wells containing KB. Association and dissociation signals are recorded, and the sensograms are fitted with the appropriate 1 : 1 binding model of the dataanalysisht v10 software.

### 2.14 Animal experiments

All mouse experiments were performed according to the Monaco animal experimentation guidelines in strict accordance with the recommendations in the Guide for the Care and Use of Laboratory Animals. Our experiments were approved by our internal ethic committee (License numbers PEA 46, 49, 51, 61).

### 2.15 *In vivo* permeability assay

Six‐week‐old female BALB/cJRj mice (Janvier Labs, Le Genest‐Saint‐Isle, France) (*n* = 10) were injected intravenously with 1% Evans blue dye (100 μL). PBS (*n* = 5) or VEGF_222/NF_ (*n* = 5) (500 ng·mL^−1^) was then injected intradermally into the right or left ear using a 30‐gauge needle (10 μL). Each mouse received two injections (PBS in one ear and VEGF_222/NF_ in the other). Twenty minutes later, the animals were euthanized, and the dye was extracted from the ears in formamide at 56 °C for 48 h. The intensity of the reaction was quantified by evaluating the absorbance of the samples at 620 nm. The ears were dried with 100% ethanol and weighed.

### 2.16 *In vivo* plug assay

PBS (*n* = 5) or VEGF_222/NF_ (*n* = 5) (200 ng) was embedded in Matrigel^®^ growth factor reduced and injected subcutaneously into the right and left flank of female NMRI mice (Janvier Labs, 2 plugs per mouse). Plugs were removed after 2 weeks for quantification of the haemoglobin.

### 2.17 Tumour experiments

ACHN‐LacZ and ACHN‐VEGF_222/NF_ cells were injected subcutaneously into the flanks of female BALB/c‐nu immunodeficient mice (*n* = 10 per condition, Janvier Labs). The mice were euthanized at the end of the experiment, and tumours were photographed with a Zeiss AXIO Zoom (Dublin, CA, USA). V16 microscope at 4× magnification. The diameter of the afferent tumour blood vessels was measured using imagej software and analysed at the first, second and third quartiles. For therapy, polyclonal mouse anti‐VEGF_XXX/NF_ antibodies were prepared after immunization of mice with the peptides DVTSRGGEPGRRKE and SGFREPDLSPGKTD. 786‐O tumour‐bearing female BALB/c‐nu mice were treated weekly with anti‐KLH antibodies (*n* = 6, KLH), anti‐VEGF_XXX/NF_ (*n* = 5, 5 mg·kg^−1^) or bevacizumab (*n* = 5, 5 mg·kg^−1^), and tumours were measured for 72 days.

### 2.18 Zebrafish studies

All animal experiments were approved by the Northern Stockholm Experimental Animal Ethical Committee. ACHN‐LacZ‐ and ACHN‐VEGF_222/NF_‐labelled cells (1,1‐dioctadecyl‐3,3,3,3‐tetramethylindocarbocyanine perchlorate [DiI, Fluka, Germany]) were injected into the perivitelline cavity of zebrafish embryos (crossed in the Karolinska zebrafish core facility). The injected embryos were kept at 28 °C and examined after 24, 48 and 72 h to monitor metastasis using a fluorescence microscope (Nikon Eclipse C1) as previously described [41].

### 2.19 Patient analysis

The study population included 47 patients with metastatic ccRCC treated with sunitinib from the SUVEGIL (NCT00943839) and TORAVA (NCT00619268) prospective trials. All patients gave a written informed consent. Blood samples were collected before the start of treatment (T0). PFS and OS were calculated for patient subgroups with VEGFA or VEGF_XXX/NF_ plasma levels (ELISA) that were below or above the third quartile value. We also had access to 60 paraffin‐embedded tumour samples from non‐metastatic patients from the UroCCCR network (NCT03293563). The presence of VEGF_XXX/NF_ was analysed by qPCR. The study methodologies conformed to the standards set by the Declaration of Helsinki.

### 2.20 Statistics

Statistical analysis was performed using graphpad prism 8. Data were expressed as mean ± SEM and were compared using an unpaired Mann–Whitney test for between‐group analysis. Comparison of tumour growth between groups was performed using two‐way ANOVA corrected for multiple comparison using Tukey's test. For patients: Student's *t*‐test was used for comparison of continuous variables, and chi‐square test or Fisher's exact test (if conditions for using of the χ^2^‐test were not met) was used for categorical variables. PFS was defined as the time between surgery and disease progression or death from any cause, censoring living patients and progression‐free patients at last follow‐up. OS was defined as the time between surgery and the date of death from any cause, censoring living patients at last follow‐up. The Kaplan–Meier method was used to generate survival curves, and analyses of the censored data were performed using Cox models. Significance was defined as *P* < 0.05.

## 3 Results

### 3.1 Identification and expression of new VEGF splice variants

In studying the anti‐angiogenics forms of VEGF, VEGF_XXXb_ [34], we looked at the DNA sequences at the junction of intron 7 and exon 8 of the human VEGF gene. A more detailed analysis revealed the presence of several AG sequences characteristic of splice acceptors. One of these sequences represents a consensus splice acceptor site located 21 bp upstream from the conventional AG splice acceptor site in the seventh intron. The presence of a consensus pyrimidine tract and a consensus “branch site” is consistent with the presence of an alternative and functional splice acceptor (Fig. S2A). The insertion of this 23 bp (including AG), defined as a new VEGF form (VEGF_/NF_), creates a new open‐reading frame that allows translation in the domain considered to be the 3′UTR of VEGF mRNA. This initial analysis was done simply by looking closely at the human sequence. An in‐depth analysis of this region of intron 7 and the start of exon 8 was performed using the Genomnis bioinformatics platform (https://hsf.genomnis.com, Fig. S2B). The Human Splicing Finder System identified all splicing elements, including both acceptor and donor splice sites, branch points and additional splicing signals (ESE and ESS). This analysis highlighted two strong branch points gcctcat (arbitrary value = 94.24) and tcctcac (arbitrary value = 98.24) upstream of the NF motif. The acceptor site of the NF exon (arbitrary value = 78.1) is a less efficient site compared to the acceptor site of exon 8 (arbitrary value = 85.83). The presence of several regulatory elements (ESS, ESE, splice acceptor sites) shows the complexity of the splicing mechanisms in this key region. It supports the hypothesis of the existence of alternative VEGF isoforms depending on the NF acceptor site. The alternative acceptor splice is located at different distances from the conventional AG in the different species. However, the frameshift in the reading frame allows translation to continue in the region corresponding to the 3′UTR. This alternative coding frame was found in GenBank for several species, including *Otolemur garnettii* (GenBank: XP_012660283.1), *Balaenoptera physalus* (GenBank: KAB0397897.1), *Camelus dromedarius* (GenBank: KAB1262026.1), *Cricetulus griseus* (GenBank: ERE90094.1) and *Chinchilla lanigera* (GenBank: XP_013372403.1). Parts of the resulting amino acid sequences (those inferred from gene analysis or those found in GenBank) are highly conserved between species but differ in length (Fig. S2C).

The mRNAs resulting from this alternative splicing encode for at least four new VEGF isoforms (Fig. 1A–D, Fig. S2D,E), although theoretically all described VEGF isoforms can contain the “NF” domain (Fig. 1A), which we have named VEGF_XXX/NF_. According to international nomenclature, removal of the signal peptide results in different VEGF_XXX/NF_: VEGF_168/NF_ (the equivalent of VEGF_111_), VEGF_178/NF_ (the equivalent of VEGF_121_), VEGF202/NF (the equivalent of VEGF145), VEGF_222/NF_ (the equivalent of VEGF_165_), VEGF240/NF, (the equivalent of VEGF183), VEGF246/NF (the equivalent of VEGF_189_), and VEGF263/NF (the equivalent of VEGF_206_). Therefore, in the rest of the text, when we talk about the general form of VEGF new form, we will use the formula VEGF_XXX/NF_. However, the recombinant VEGF new form corresponding to VEGF_165_ will be referred to as VEGF_222/NF_. Total VEGF and VEGF_XXX/NF_ mRNA were amplified by RT‐PCR (Fig. S2D) and RT‐qPCR (Fig. 1B) from human tissues and RCC cell lines (Fig. 1C and Fig. S2E) and the amplicons obtained with RNA from endothelial cells (TIME) served as reference values. VEGF_XXX/NF_ mRNAs were also detected in model cell lines of triple negative breast cancer (MDA‐MB231), medulloblastoma (DAOY) and pancreatic ductal adenocarcinoma (MiaPaca‐2, Fig. S3).

In normal tissue (Fig. S2D), the predominant amplicons for VEGF correspond to VEGF_189_, VEGF_165_ and VEGF_121_. TIME cells predominantly expressed VEGF_165_ and VEGF_121_ although the amplicons did not migrate at the expected size for unknown reasons. For VEGF_XXX/NF_, the predominant amplicons were VEGF246/NF, VEGF_222/NF_ and VEGF_178/NF_. VEGF263/NF was detected in kidney and was the only isoform detected in TIME cells. All amplicons were subcloned into plasmids and verified by sequencing.

The qPCR analyses showed that the kidney expressed the highest levels of VEGF and the lung expressed the highest levels of VEGF_XXX/_NF (Fig. 1B).

In RCC cells mutated for VHL or not [786‐O (786), ACHN, (A), Fig. S2E], the predominant amplicons for VEGF corresponded to VEGF_189_, VEGF_165_ and VEGF_121_. For VEGF_XXX/_NF, the predominant amplicons were VEGF263/NF, VEGF246/NF and VEGF_222/NF_. VEGF_178/NF_ was expressed only in 786‐O cells. Again, the sequences of the amplicons were verified by sequencing.

The qPCR analyses showed that the cells mutated for VHL, with the exception of CAKI‐2 expressing a wild‐type form of VHL, expressed high levels of VEGF. 786‐O cells mutated for VHL expressing only HIF2α, expressed the highest levels. With the exception of RCC10, cells mutated for VHL (RCC4, 786‐O, A498) expressed high levels of VEGF_XXX/_NF. Again, 786‐O showed the highest levels. Re‐expression of VHL (786‐VHL) decreased VEGF and VEGFNF levels suggesting that both isoforms are regulated by hypoxia‐inducible factors (HIF1 and/or HIF2, Fig. 1C). A summary table recapitulates the expression of VEGF and VEGF_XXX/NF_ in the different cells and tissues (Fig. 1D).

Two polyclonal anti‐VEGF_XXX/NF_ antibodies were raised against two conserved epitopes (DVTSRGGEPGRRKE and SGFREPDLSPGKTD, Fig. S4A). The preimmune crude sera from rabbits immunized with peptide 1 or peptide 2 detected non‐specific proteins. However, the specific crude sera detected an additional band only in conditioned media from cells transfected with an expression vector encoding VEGF_222/NF_, but not in cells transfected with an empty vector (Fig. S4B). These antibodies were not reactive to purified VEGF_165_ or VEGF_165b_ (Fig. S4C). They did not detect protein in the conditioned media or in lysate of cells transfected with an empty expression vector or with an expression vector encoding VEGF_165_ (Fig. S4C,D). However, they detected a 30‐kDa protein in the conditioned medium and to a lesser extent in the lysate of cells transfected with an expression vector encoding VEGF_222/NF_ protein (Fig. S4D). These experiments demonstrate the specificity of these antibodies for VEGF_XXX/NF_. This result also indicates that almost VEGF_XXX/NF_ produced is secreted (maximum amounts in conditioned medium). Rabbit anti‐VEGF_XXX/NF_ antibodies (clone #2) were used to detect the presence of VEGF_XXX/NF_ in conditioned media from normal and tumour cells. VEGF_XXX/NF_ was undetectable in TIME cells and was detected in the conditioned media of almost all RCC cell lines except ACHN, with the highest levels found in 786‐O and A498 cells (Fig. 1E). These results strongly suggest the existence of a new VEGF splice variant.

### 3.2 VEGF_222_ _/NF_ promotes angiogenesis

**Fig. 2:**
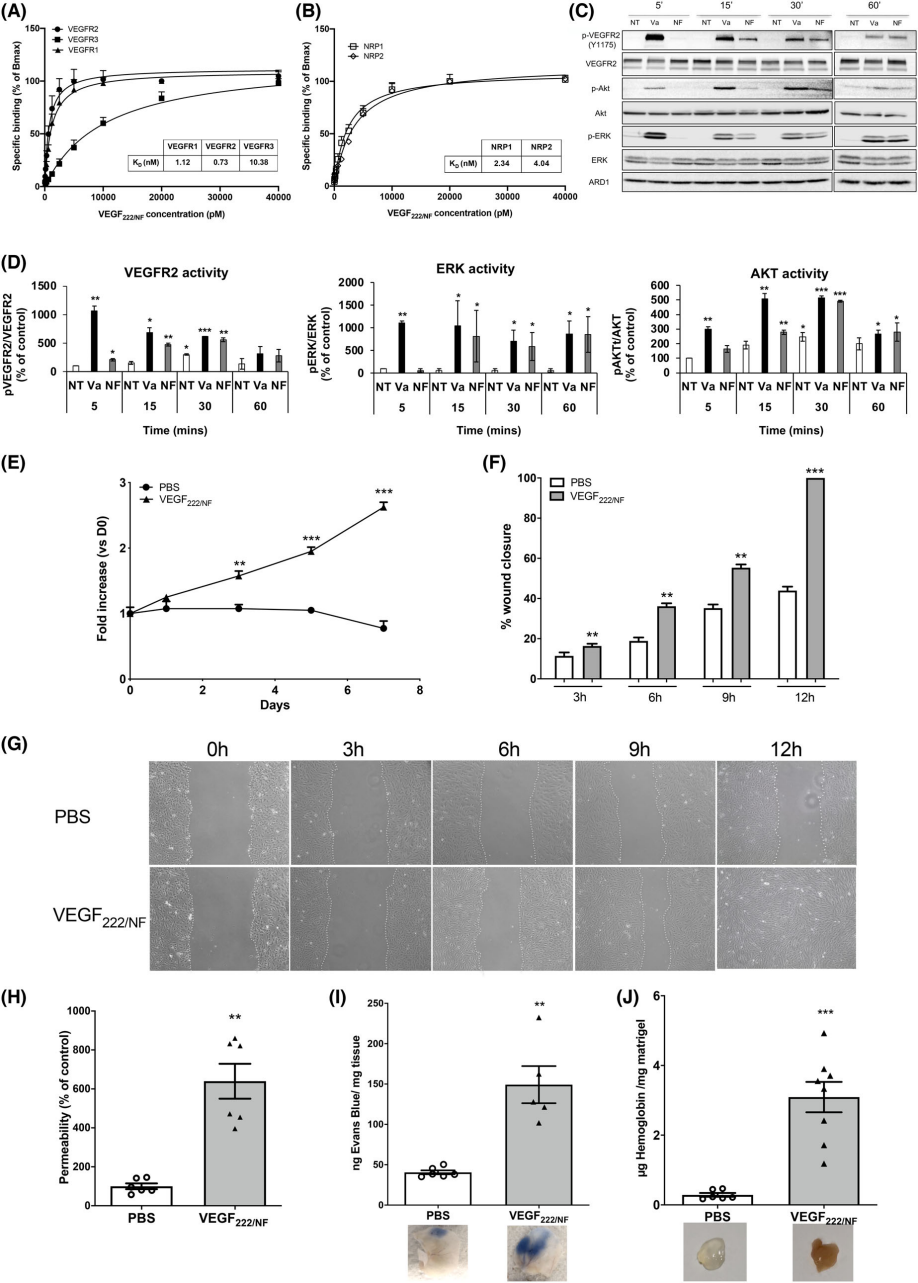
VEGF_222/NF_ binds VEGF‐receptors and co‐receptors and stimulates angiogenesis. (A, B) Specific binding of VEGF_222/NF_ to VEGFRs (VEGFR1, VEGFR2, VEGFR3) (A) and to NRPs (NRP1, NRP2) (B). (C) VEGF_222/NF_ induces phosphorylation of VEGFR2 and activation of downstream signalling pathways AKT and ERK. Confluent monolayers of TIME/endothelial cells were kept serum starved for 2 h and then treated with VEGF_165_ (Va, 100 ng·mL^−1^) or VEGF_222/NF_ (NF, 100 ng/mL) for the indicated times. Figure shows representative images from three independent experiments. (D) Quantification of VEGFR2, ERK and AKT activity measure as a ratio between the amounts of phosphorylated and unmodified protein. The untreated (NT) condition at 5 min was used as reference, *n* = 3, *P < 0.05; **P < 0.01; ***P < 0.001. (E) Cell proliferation assay of serum‐starved endothelial cells (TIME) treated with VEGF_222/NF_ (100 ng·mL^−1^) or not. Cells were counted for 7 days. (F–G) Wound scratch assays performed with serum‐starved TIME cells treated with VEGF_222/NF_ (100 ng·mL^−1^). Wound closure was determined at 3‐, 6‐, 9‐ and 12‐h after treatment. (H) *In vitro* permeability assay. Monolayers of serum‐depleted TIME cells were treated with PBS or VEGF_222/NF_ (100 ng·mL^−1^) for 30 min. Permeability was assessed by streptavidin‐HRP and TMB substrate staining. (I) *In vivo* permeability assay. Mice were injected intravenously with Evans blue, followed by PBS or VEGF_222/NF_ (500 ng·mL^−1^). The amount of Evans blue was determined colorimetrically in mouse ears (upper panel). Representative photos of vascular leakage induced by PBS or VEGF_222/NF_ (bottom panel). (J) *In vivo* plug assay. Mice were injected with a low concentration of Matrigel® containing PBS or VEGF_222/NF_ (1 μg·mL^−1^) and the level of haemoglobin content was measured 15 days after implantation. Representative photos of the Matrigel® plug 15 days after implantation (lower panel). Data were expressed as mean ± SEM. **P 0.01, ***P < 0.001 vs PBS (two‐way ANOVA was used to assess statistical difference for cell proliferation and a Mann–Whitney test for the permeability and plug assays). Results of *in vitro* experiments are presented as the mean ± SEM. All experiments were performed with at least three biological duplicates (*n* = 3) for each group in triplicate.

The ability of VEGF_222/NF_ to specifically bind VEGFRs and co‐receptors was first investigated by saturation binding experiments. VEGF_222/NF_ binds VEGFR1 and VEGFR2 with an affinity in the nanomolar range, with a KD of 1.12 and 0.73 nm, respectively (Fig. 2A). VEGF_222/NF_ was also found to bind the VEGF co‐receptors neuropilin 1 and 2 (NRP1 and NRP2) with comparable affinity (Fig. 2B). Despite a lower affinity, VEGF_222/NF_ also binds VEGFR3 (KD = 10.38 nm). We have attempted to confirm these results using the Octet method, which requires modifications to allow the receptors/coreceptors to bind to specific biosensors. By this independent method, we confirmed binding of VEGF_222/NF_ to VEGFR1 and NRP1. However, we could not confirm binding to VEGFR3 and NRP2. VEGF_165_ and VEGFC were used as controls in the same experiment. VEGF_165_ bound with a higher affinity to VEGFR2, which is not consistent with the literature showing better affinity for VEGFR1 [42]. According to the Octet provider, this result is probably due to the slow dissociation of VEGF_165_ from VEGFR2. Although VEGFC binds with a high affinity to VEGFR3, it does not bind to NRP2, which is not consistent with the literature [43] (Fig. S5A,B). Therefore, we were more confident with the ELISA method as it does not require modification of the receptors/coreceptors that could affect the binding of the ligands. These results prompted us to investigate the effects of VEGF_222/NF_ on physiological angiogenesis. VEGF_222/NF_ induced sustained phosphorylation/activation of VEGFR2 and subsequent ERK and AKT activation in endothelial cells (ECs) (Fig. 2C,D). These results are consistent with the pro‐proliferative effects of VEGF_222/NF_ in ECs (Fig. 2E). VEGF_222/NF_ stimulated the migration of ECs in wound healing assays (Fig. 2F,G). VEGF_222/NF_ also showed a pro‐permeable activity inhibited by the VEGFR1/2/3 inhibitor axitinib (Fig. S6A).

In these experiments, axitinib inhibited the VEGF_222/NF_‐dependent activation of VEGFR2 and ERKs (Fig. S6B).The beneficial effects of VEGF_222/NF_ on the permeability of ECs were confirmed *in vivo* by measuring the extravasation of Evans blue dye (Fig. 2I). This pro‐permeability activity was threefold higher compared to control conditions (*P* < 0.01). Matrigel^®^ plug assays showed that the haemoglobin content of VEGF_222/NF_ plugs was higher compared to control conditions (Fig. 2J). These experiments demonstrated that VEGF_222/NF_ has pro‐angiogenic properties in a physiological context.

### 3.3 VEGF_222_ _/NF_ promotes survival and proliferation of ccRCC cells

The role of VEGF in cancer is not limited to angiogenesis and vascular permeability. VEGF‐mediated signalling occurs in tumour cells and contributes to important aspects of tumourigenesis. The proliferative effect of VEGF_222/NF_ was first investigated in ACHN cells, in which the VEGF_XXX/NF_ protein is undetectable by ELISA (Fig. 1G). Overexpression of VEGF_222/NF_ in ACHN cells was first confirmed by RT‐qPCR analysis (Fig. S7A) and ELISA (Fig. S7B). Overexpression of VEGF_222/NF_ did not affect the levels of another splice form of VEGF, VEGF_121_ (Fig. S7A) indicating that overexpression of VEGF_222/NF_ did not alter overall VEGF expression. VEGF_222/NF_ stimulated proliferation of ACHN cell (*P* < 0.001, Fig. S7C). Two independent shRNAs directed against VEGF_XXX/NF_ (shRNA NF#1 and NF#2) downregulated the expression of their respective targets in ACHN and 786‐O cells (Fig. S7D,E). Downregulation of VEGF_XXX/NF_ triggered cell death in clonogenicity assays (Fig. S7F). Since VEGF exerts autocrine proliferation loops via NRP1 and NRP2, we tested the effect of downregulation of both genes by shRNA (Fig. S7G). Proliferation of ACHN expressing VEGF_222/NF_ was inhibited mainly by reducing the expression of NRP1 (Fig. S7H) and to a lesser extent by reducing the expression of NRP2 (Fig. S7I). VEGF_222/NF_ thus stimulated an autocrine proliferation loop involving at least the NRP1/2 signalling pathways. These results strongly suggest that VEGF_XXX/NF_ plays a key role in ccRCC cell survival and proliferation.

### 3.4 VEGF_222_ _/NF_ promotes tumour growth and induces tumour angiogenesis and lymphangiogenesis

**Fig. 3:**
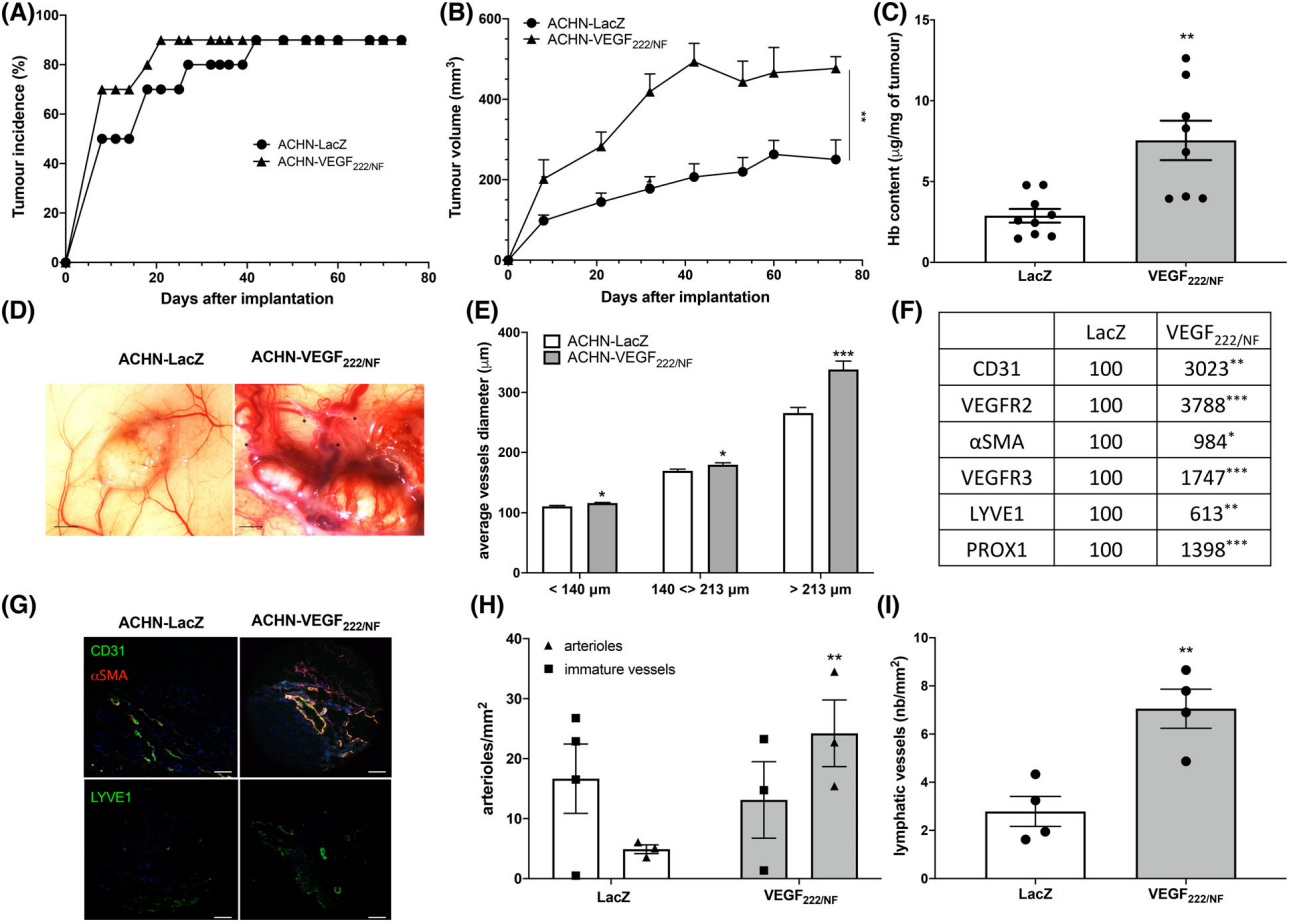
VEGF_222/NF_ promotes tumour growth and induces tumour angiogenesis, lymphangiogenesis and vascular maturation. (A) Determination of tumour incidence in nude mice with ACHN‐LacZ, ACHN‐VEGF_222/NF_ (*n* = 10 per group) tumours. (B) Tumour growth curves of ACHN‐LacZ and ACHN‐VEGF_222/NF_ cells. Tumour volume was measured with calliper every week for 70 days (*n* = 10 per group). (C) Relative haemoglobin content of ACHN‐LacZ and ACHN‐VEGF_222/NF_ tumours (*n* = 10 per group). (D) Representative photographs of ACHN‐LacZ and ACHN‐VEGF_222/NF_ tumours (*n* = 10 per group) with blood vessels and lymphatic vessels (indicated by black stars, scale bar: 5 mm). (E) Average diameter of peri‐tumoural vessels (*n* = 10 per group) of the first (< 140 μm), second (between 140 and 213 μm diameter) and third quartiles (> 213 μm). (F) RT‐qPCR analysis of angiogenic and lymphangiogenic genes expressed in ACHN‐LacZ (*n* = 5) and ACHN‐VEGF_222/NF_ tumours (*n* = 5). (G) Immunofluorescence detection of CD31 and α‐SMA (upper panel) and LYVE1 (lower panel) in ACHN‐LacZ (*n* = 5) and ACHN‐VEGF_222/NF_ (*n* = 5) tumours. Scale bar: 200 μm. (H) Number of capillaries (CD31^+^) and arterioles (CD31^+^, α‐SMA^+^) vessels in ACHN‐LacZ (*n* = 4) and in ACHN‐VEGF_222/NF_ (*n* = 4) tumour sections. (I) Number of lymphatic vessels (LYVE1^+^) in ACHN‐LacZ (*n* = 4) and in ACHN‐VEGF_222/NF_ (*n* = 4) tumour sections. *P < 0.05, **P < 0.01, ***P < 0.001 vs LacZ (Mann–Whitney). Results are presented as the mean ± SEM.

The pro‐tumour activity of VEGF_XXX/NF_ was determined by generating experimental tumours with ACHN‐VEGF_222/NF_ cells in immunodeficient mice. At day 21, an incidence of 90% was achieved in the ACHN‐VEGF_222/NF_ group, compared to 70% in the control group (Fig. 3A). Tumours generated with ACHN‐VEGF_222/NF_ were 2.5‐fold larger compared to ACHN‐LacZ control tumours (Fig. 3B). Tumour vascularization, as measured by testing the haemoglobin content of the tumours, was 2.5‐fold higher in ACHN‐VEGF_222/NF_ compared to ACHN‐LACZ tumours (*P* < 0.01, Fig. 3C). Peri‐tumour vascularization and vessel diameter were higher in the ACHN‐VEGF_222/NF_ group than in the ACHN‐LacZ group (Fig. 3D). Quantification of blood vessel diameter confirmed this observation; the average diameter of blood vessels reaching the ACHN‐VEGF_222/NF_ tumours was higher than the diameter of vessels in the control group (Fig. 3E). In addition to the blood vessel network, an unexpectedly dense lymphatic vessel network reached the ACHN‐VEGF_222/NF_ tumours (black stars, Fig. 3D). qPCR analysis showed that VEGF_222/NF_ overexpression correlated with increased levels of CD31, VEGFR2 and αSMA (angiogenesis markers) and VEGFR3, LYVE1 and PROX1 (lymphangiogenesis markers; Fig. 3F). This observation suggests that VEGF_222/NF_ stimulates the development of arterioles covered with αSMA‐positive pericytes and the development of an extensive lymphatic network. Immunofluorescence labelling with anti‐CD31 and anti‐αSMA confirmed a higher number of arterioles (CD31^+^ and αSMA^+^) for VEGF_222/NF_ compared with control tumours (*P* < 0.05, Fig. 3G,H). LYVE1 labelling also confirmed a denser lymphatic network in VEGF_222/NF_ tumours compared with the control group (*P* < 0.05, Fig. 3I). VEGF_222/NF_ induced the proliferation of human dermal lymphatic endothelial cells (HDLECs) and induced phosphorylation of VEGFR3 (Fig. S8A,B). These results suggest that VEGF_222/NF_ is a potent pro‐angiogenic factor that also stimulates lymphangiogenesis.

### 3.5 VEGF_222_ _/NF_ promotes distant metastasis in zebrafish

**Fig. 4:**
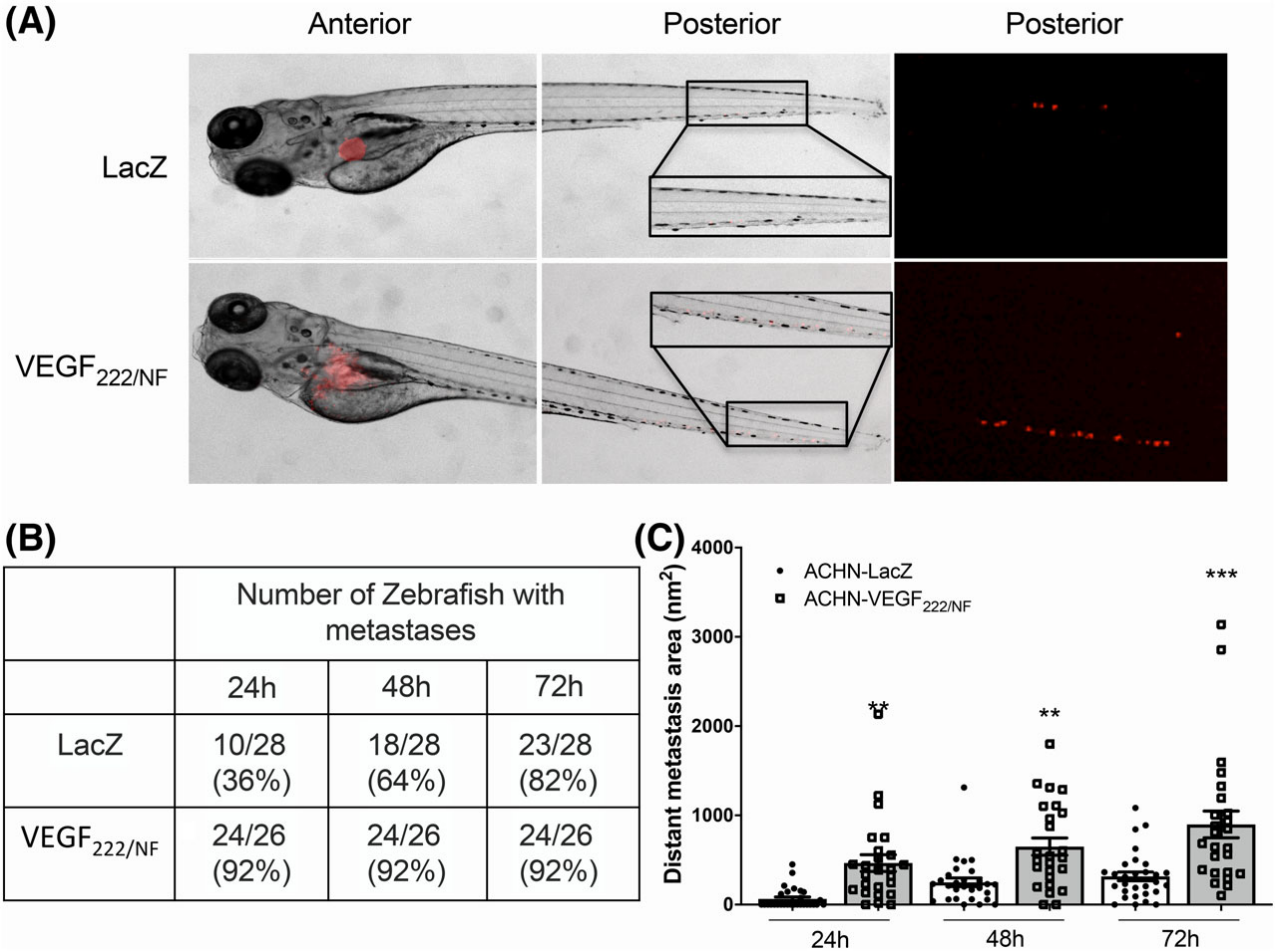
VEGF_222/NF_ is associated with the spread of metastases in zebrafish. (A) Representative photographs of zebrafish embryos (*n* = 35) injected with red‐DiD‐labelled ACHN‐LacZ and ACHN‐VEGF_222/NF_ cells into the perivitelline space. The zebrafish embryos were examined for tumour metastases using a fluorescence microscope (middle and right panels). (B) Table showing the number of zebrafish embryos with scattered tumour foci in the tails. (C) The areas of metastasis in the tails of zebrafish embryos were quantified 24, 48 and 72 h after injection of cells. **P < 0.01, ***P < 0.001 vs LacZ (two‐way ANOVA). Results are presented as the mean ± SEM. Experiments were performed with at least three biological duplicates (*n* = 3) for each group in triplicate.

Unfortunately, none of the human cell lines used in our study developed metastases when xenotransplanted into immunodeficient mice. The zebrafish is a unique experimental model for studying cancer development and metastasis [44]. To further investigate the role of VEGF_222/NF_ in tumour angiogenesis and metastasis, we next examined the ability of VEGF_222/NF_‐expressing tumour cells to spread from the injection site, extravasate from the bloodstream and become established metastases in the tails of zebrafish. ACHN‐LacZ and ACHN‐VEGF_222/NF_ cells were xenografted into the perivitelline zone, and the metastatic foci resulting from cell extravasation from the bloodstream and their proliferation in the tail (foci with more than one cell) were examined after 24, 48 and 72 h (Fig. 4A,B). The ACHN‐VEGF_222/NF_ group was found to have a significant increase in the spread of tumour cells compared to the control group at all time points examined (Fig. 4B). Although control cells can form distant metastases even after a longer period of time, the size of metastatic foci formed with ACHN‐VEGF_222/NF_ cells was larger. These results suggest that VEGF_222/NF_ promotes the spread of metastases.

### 3.6 Inhibition of VEGF_222_ _/NF_ delayed the growth of experimental RCC

**Fig. 5:**
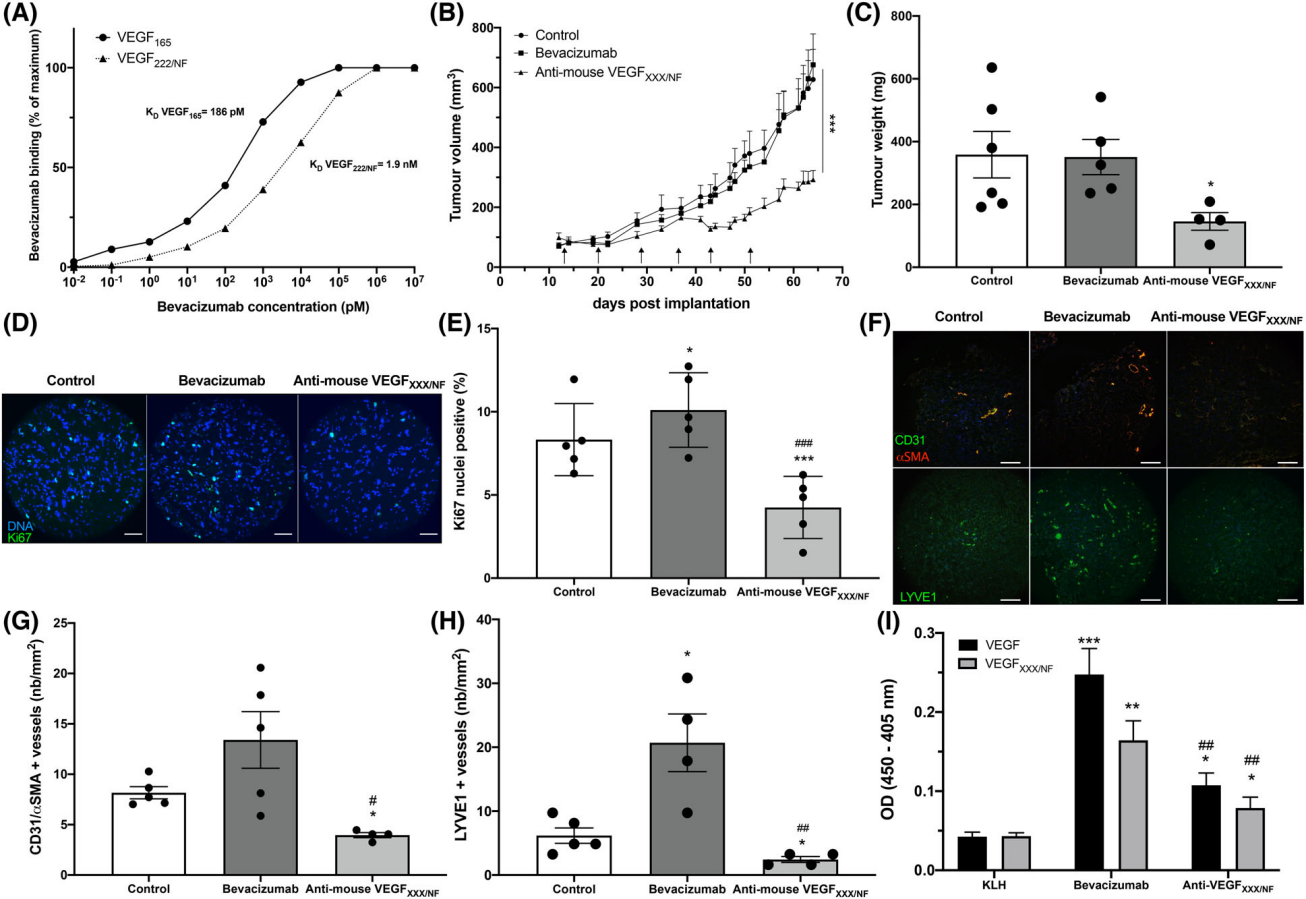
Inhibition of VEGF_XXX/NF_ delays the growth of experimental RCC tumours. (A) The affinity of bevacizumab for VEGF_165_ and VEGF_222/NF_ was determined by ELISA, *n* = 3. (B) The growth curve of experimental tumours generated with 786‐O cells after treatment with anti‐KLH (*n* = 6), anti‐VEGF_XXX/NF_ (*n* = 5) and bevacizumab (*n* = 5). (C) Weight of 786‐O tumours at the end of the experiment. (D–E) Quantification of Ki67‐positive cells in 786‐O tumours. Cell proliferation was detected by Ki67 immunofluorescence labelling (green) and Hoechst33342 nuclear DNA counterstaining (blue). Scale bar: 50 μm. (F) Immunofluorescence detection of CD31, α‐SMA (upper panel) and LYVE1 (lower panel) in 786‐O tumours treated with control, anti‐VEGF_XXX/NF_ antibodies or bevacizumab. Scale bar: 200 μm. (G) Number of mature (CD31^+^, α‐SMA^+^) vessels in the different tumour sections. (H) Number of lymphatic vessels (LYVE1^+^) in the tumour sections. *P < 0.05, ***P < 0.001 vs control, ^#^ *P* < 0.01, ^##^ *P* < 0.01, ^###^ *P* < 0.001 vs bevacizumab (one‐way ANOVA). (I) Plasma levels of VEGF_XXX/NF_ and VEGF increased in the bevacizumab‐treated group. ELISA of plasma levels of VEGF and VEGF_XXX/NF_ in mice with 786‐O tumours treated with bevacizumab or KLH or anti‐mouse VEGF_XXX/NF_ antibodies. *P < 0.05, **P < 0.01 vs KLH, ^#^ *P* < 0.05 vs bevacizumab (one‐way ANOVA).

Bevacizumab has previously been shown to be less effective if tumours expressing VEGF_XXXb_ isoforms [45]. Therefore, we hypothesized that the presence of VEGF_XXX/NF_ might also influence the efficacy of bevacizumab. We showed that bevacizumab has a 10‐fold lower affinity for VEGF_222/NF_ compared to VEGF_165_ (Fig. 5A). Polyclonal antibodies directed against VEGF_XXX/NF_ were generated in mice (Anti‐mouse VEGF_XXX/NF_) by injecting the same peptides used to generate polyclonal antibodies in rabbits, and their specificity was characterized. They recognized only recombinant VEGF_222/NF_ by immunoblot (Fig. S9A) or by ELISA (Fig. S9B). Anti‐mouse VEGF_XXX/NF_ antibodies significantly slowed the growth of experimental tumours generated with 786‐O cells by 56%, while the size of tumours in bevacizumab‐treated mice was similar to that of the control group, as previously described [46] (Fig. 5B). This result was consistent with a 60% decrease in the weight of tumours from mice treated with the Anti‐mouse VEGF_XXX/NF_ antibodies (Fig. 5C). The number of proliferative Ki67‐positive cells decreased sharply in these tumours but not in the tumours of mice treated with bevacizumab (Fig. 5D,E). In addition to their antiproliferative effect, anti‐mouse VEGF_XXX/NF_ antibodies also decreased the number of CD31^+^/αSMA^+^ vessels within the tumour (Fig. 5F,G). In addition, anti‐mouse VEGF_XXX/NF_ antibodies decreased the number of lymphatic vessels while bevacizumab stimulated their development, as we have described previously [47, 48] (Fig. 5F–H). Furthermore, a threefold increase in plasmatic levels of VEGF_XXX/NF_ was observed in mice treated with bevacizumab (Fig. 5I). These results highlight the importance of specific VEGF_XXX/NF_ inhibition for the treatment of RCC.

### 3.7 VEGF_XXX_ _/NF_ is synonymous with poor prognosis in metastatic ccRCC patients and predicts response to sunitinib but not to bevacizumab

**Fig. 6:**
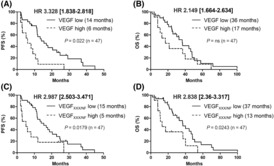
VEGF_XXX/NF_ is associated with poor prognosis in metastatic ccRCC patients and predicts response to sunitinib. Levels of VEGF and VEGF_XXX/NF_ were assessed in plasma (just before sunitinib treatment) from 47 metastatic ccRCC patients (SUVEGIL and TORAVA cohorts). The third quartile was used as the cut‐off value for determining the patient group, that is, 4500 and 3000 pg·mL^−1^ for VEGF and VEGF_XXX/NF_, respectively. Correlation of high or low plasma levels of VEGF (A, B) or VEGF_XXX/NF_ (C, D) with PFS and OS during first‐line treatment with sunitinib is shown. The Kaplan–Meier method was used to generate survival curves, and Cox models were used to analyse censored data. Statistical significance (*P* values) and hazard ratios are indicated in the corresponding panels.

The deleterious effects of VEGF_222/NF_ in immunodeficient mice and zebrafish prompted us to analyse the prognostic significance of its plasmatic levels compared with plasmatic VEGF levels in a cohort of 47 metastatic (M1) ccRCC patients treated with sunitinib and in a cohort of 30 patients treated with bevacizumab plus interferon alpha or temsirolimus [49]. The clinical characteristics of these patients are shown in Figs S10 and S11. In patients treated with sunitinib, VEGF and VEGF_XXX/NF_ were undetectable in the plasma of the same five patients and high expression (above the third quartile) was detected in the plasma of the same eight patients. VEGF and VEGF_XXX/NF_ were not systematically high or low in the other samples from the same patients. The same trend (no systematic equal expression level of VEGF and VEGF_XXX/NF_) was observed for the plasma of patients treated with bevacizumab. The PFS of patients treated with sunitinib was significantly shortened in the high VEGF and high VEGF_XXX/NF_ groups (Fig. 6A–C). Importantly, VEGF (Fig. S12A,B) and VEGF_XXX/NF_ (Fig. S13C,D) plasmatic levels did not affect PFS and OS of bevacizumab‐treated patients regardless of the combination with interferon or temsirolimus.

Therefore, these results suggest that VEGF, as previously described [50], and VEGF_XXX/NF_ should be considered equivalent predictive markers for response to sunitinib but not to bevacizumab. Furthermore, patients with high plasma levels of both VEGF_XXX/NF_ and VEGF had the shortest PFS (Fig. S13). This result suggests that the levels of both VEGFs should be tested to stratify patients eligible for sunitinib therapy. While VEGF had no significant effect on OS, high expression of VEGF_XXX/NF_ was equivalent to a shorter OS in M1 patients (Fig. 6B,D). Therefore, VEGF_XXX/NF_ is a more robust prognostic marker compared to VEGF.

To further demonstrate the clinical relevance of the VEGF_XXX/NF_ splice variants in ccRCC, it was important to detect its presence in tumour tissue. We only had access to tumour samples from 60 non‐metastatic patients. The presence of VEGF_XXX/NF_ was detected by qPCR in 100% of the tumours examined but with a different range of expression (Fig. S14). Although these results need to be confirmed again in an independent cohort, they demonstrate the relevance of VEGF_XXX/NF_ in patient samples.

## 4 Discussion

Not all the secrets of VEGF have been revealed. Thirty years after its discovery, we describe new isoforms of VEGF that arise from an unknown alternative splicing. All splice variants described so far result in a minor change in the protein sequence with insertion of 6 amino acids for the alternative exons 8a or 8b, 12 amino acids for exon 7b, 17 amino acids for exon 6b, 25 amino acids for exon 6a and 32 amino acids for exon 7A [4]. The new isoforms described in this study corresponded to an addition of 64 amino acids. Additional splice acceptor sites are present in the different VEGF introns. Two hundred and twenty‐nine “AG” consensus sites are present only in the first intron, and several in the other introns, multiplying the potential number of splicing events in the VEGF gene. This potential multiplication of VEGF isoforms opens up a new area of research in the field of VEGF. Modification to the C‐terminal part of the protein, the NRP‐binding domain of conventional VEGF is replaced by an alternative sequence. This feature has already been described for the VEGF_XXXb_ isoforms, in which the CDKPRR sequence, the NRP1‐binding domain, was modified to SLTRKD [34]. Modification of this C‐terminal part has also been described for VEGF‐Ax, a form of VEGF that is generated by translation through the stop codon [35]. Anti‐angiogenic properties were first described for the VEGF_XXXb_ and VEGF‐Ax isoforms. Although less potent compared to VEGF, both isoforms showed pro‐angiogenic properties [36, 51]. According to these results, we expected that altering the C‐terminal part of VEGF_XXX/NF_ should lead to the same controversial situation. VEGF_222/NF_, the predominant isoform of VEGF_XXX/NF_, stimulated proliferation of endothelial cells. The resulting blood vessels in experimental tumours resembled normal and functional arterioles covered with pericytes. This property of VEGF_222/NF_ favours tumour vascularization and promotes tumour growth. As the tumour progresses, the VEGF_222/NF_‐dependent functional vascular network thus becomes a major player in tumour cell proliferation and spreading. Unexpectedly, VEGF_222/NF_ also promoted the development of a lymphatic network, which should also promote spread of metastases. So, in advanced stages, VEGF_222/NF_ may promote tumour aggressiveness. Like VEGF, VEGF_222/NF_ exerts its deleterious effects by promoting tumour vascularization but also by stimulating tumour cell proliferation via autocrine loops. Although several tumour cells express VEGF and its receptors VEGFR1‐3 together [52], ccRCC cells do not express VEGFRs [53]. Instead, they express NRP1 and NRP2 which mediate autocrine proliferation loops involving VEGF and VEGFC [53, 54]. NRP1 and, to a lesser extent, NRP2 are interesting signalling partners of VEGF_222/NF_, as their downregulation reduces VEGF_222/NF_‐dependent proliferation. The CDKPRR motif is not present in the C‐terminal part of the VEGF_222/NF_ sequence. However, a PGRRK motif is highly conserved between species. Proteolytic cleavage could result in a new basic‐rich domain that allows NRP1/2 binding.

More importantly, cells overexpressing VEGF_222/NF_ became dependent on this autocrine loop, which exerts proliferation but also pro‐survival properties. VEGF has also been described as a driver of immune tolerance by stimulating the expression of immune checkpoints on the surface of T cells through the stimulation of VEGFR2 [55]. We hypothesize that VEGF_222/NF_ will have the same effects as it stimulates VEGFR2. Therefore, we expect that targeting VEGF_222/NF_ should inhibit the three major hallmarks of cancer: tumour cell proliferation, angio/lymphangiogenesis and immune tolerance at an advanced/metastatic stage of tumour development. Our results clearly show that VEGFR2 and NRP1/2 are involved in the VEGF_222/NF_‐dependent signalling pathway. Furthermore, we have shown that a lymphatic network developed in experimental tumours generated with VEGF_222/NF_‐overexpressing ACHN cells. *In vitro* experiments showed that VEGF_222/NF_ exerted a direct effect on lymphatic endothelial cells. To our knowledge, this is the first VEGF isoform to activate lymphangiogenesis via this receptor.

Inhibition of VEGF_XXX/NF_ by specific antibodies in experimental RCC significantly reduced tumour growth, whereas this was not the case with bevacizumab. The antitumour effects of anti‐VEGF_XXX/NF_ antibodies depended on reduced blood vessel density and reduced proliferation of tumour cells. Compared to the modest effects of bevacizumab, targeting VEGF_XXX/NF_ appears to be a relevant therapeutic strategy.

Clinically, the presence of plasmatic VEGF_XXX/NF_ correlates with poor prognosis in metastatic ccRCC, which is consistent with the effect of VEGF_XXX/NF_ in experimental tumours. Generalization of this concept to different tumour types is now possible thanks to the availability of our homemade ELISA. Bevacizumab, the anti‐VEGF antibody, failed to increase the OS of ccRCC and breast cancer patients, resulting in the FDA losing approval for both cancers. The presence of VEGF_XXX/NF_ and classical VEGF is one of the explanations for the failure of bevacizumab to treat both cancers. The presence of VEGF_XXXb_ lowers the efficacy of bevacizumab in colorectal cancer [45]. These results have been attributed to the anti‐angiogenic role of VEGF_XXXb_ [34]. However, it is also possible that a modification of the C‐terminal part of VEGF alters the affinity for its target. VEGF acts as a dimer involving the cysteine residue of the outermost C‐terminal part “CDKPRR,” which is lost in VEGF_XXX/NF_. The change in the dimer conformation caused by the absence of the disulphide bridge alters the three‐dimensional structure and recognition by bevacizumab. Alteration of the 3D conformation of VEGF_XXX/NF_ homodimers or of VEGF/VEGF_XXX/NF_ heterodimers does not lead to optimal recognition by bevacizumab. Here, we show that bevacizumab has a 10‐fold higher affinity for VEGF_165_ compared to VEGF_222/NF_ confirming this hypothesis. Furthermore, we showed that bevacizumab increased the plasmatic VEGF_XXX/NF_ levels of tumour‐bearing mice. Such an increase is consistent with the reduced anti‐tumour activity of bevacizumab. VEGF_XXX/NF_ levels need to be determined for optimal bevacizumab doses which will be established in early‐phase studies. Before bevacizumab was approved, different doses were tested: 5, 7.5, 10 and 15 mg·kg^−1^. Depending on the type of cancer, different doses were approved to achieve maximum therapeutic response and limit toxicity. For example, the dose of 10 mg·kg^−1^ in combination with interferon alpha was approved for ccRCC [56]. Higher concentrations of bevacizumab are more toxic, but should have been more effective due to the simultaneous inhibition of VEGF and VEGF_XXX/NF_. A comparable situation concerns the use of gefitinib to treat lung cancer patients. The drug is effective at a daily dose of 250 mg only in patients with certain mutations in the EGF receptor. Higher doses, which inhibit both wild‐type and mutated forms of the EGF receptor, cannot be administered because of toxicity [57]. Since VEGF_XXX/NF_ expression affects survival in advanced stage ccRCC, the development of a specific antibody is worth considering. Detection of VEGF and VEGF_XXX/NF_ in blood would serve as a companion test to select patients eligible for treatment with anti‐VEGF_XXX/NF_ alone or in combination with bevacizumab. In the cohort of patients we studied, we found an association between high VEGF_XXX/NF_ levels and sensitivity to sunitinib but not to bevacizumab. Although this result suggests that VEGF_XXX/NF_ is a predictive marker for the efficacy of sunitinib but not bevacizumab, it needs to be confirmed in independent patient cohorts. In the SUVEGIL and TORAVA clinical trials, we examined samples after the second, third and fourth cycles of sunitinib treatment. The variation in values between patients was large. Therefore, no true statistical significances were found during the course of treatment. As we have previously published for other biomarkers associated with these studies, all of the markers we examined were differentially expressed at diagnosis and these differences correlated with survival (OS, PFS) [49, 58, 59]. In this study, we only investigated VEGF‐dependent neoplasms. However, VEGF is also involved in several pathologies, particularly ocular diseases such as vascular age‐related macular degeneration (vAMD), for which anti‐VEGF is the standard treatment [60]. However, anti‐VEGF treatment is ineffective or only temporarily effective in more than 30% of patients with this pathology, and these patients go blind 2 years after a relapse. As in cancer, we suspect that the presence of VEGF_XXX/NF_ would limit the therapeutic effect of anti‐VEGF. High VEGF levels were found in patients with COVID‐19 [61]. They had severe endothelial damage of the lung and alveolar damage with infiltration of perivascular lymphocytes. The presence of a high concentration of VEGF_XXX/NF_ in the lungs may suggest that this new form could play an important role in severely infected patients.

## 5 Conclusions

We believe that our results represent an important breakthrough in the field of angiogenesis and that they explain the failure of anti‐VEGF therapies. Given the discovery of the existence of this specific isoform, we point out that much of the literature on VEGF is inaccurate or even wrong. Re‐evaluating this very important area by increasing knowledge about VEGF_XXX/NF_ could lead to new therapeutic strategies for diseases in which the VEGF/VEGF_XXX/NF_/angiogenesis axis plays an important role.

## Conflict of interest

We disclose that GP is the co‐founders of the startup Kekkan Biologics.

## Author contributions

The study was conceived and designed by JD, CM and GP. The methodology was developed by JD, CM, XH and YC. The data were acquired by JD, CM, VV, CG, XH, AK, DA, SN, J‐CB and DB. The data were analysed and interpreted by JD, CM, CG, MD, YC and DA. CM and GP wrote and reviewed the article. GP did the administrative, technical, or material support and study supervision.

6

### Peer review

The peer review history for this article is available at https://publons.com/publon/10.1002/1878‐0261.13401.

## Supporting information


**Fig. S1.** DNA and protein sequences of native and recombinant VEGF_222/NF_. (A) DNA sequence encoding the native VEGF_222/NF_ protein and (B) the native VEGF_222/NF_ protein. (C) DNA sequence encoding the optimized His‐tagged VEGF_222/NF_ sequence and (D) the corresponding protein sequence produced by ProteoGenix.

**Fig. S2.** Bioinformatic analysis of the last intron of the human VEGF gene and conservation of the protein from the resulting splicing event in different mammals. (A) Possible splicing events of VEGF pre‐mRNA and the resulting C‐terminal specific sequence of VEGF_XXX/NF_ and VEGF_165_. (B) Bioinformatic analysis of the intron 7 exon 8 part with ESS and ESE motifs. (C) Conservation of the C‐terminal sequence of VEGF_XXX/NF_ between species. HS: *Homo sapiens*; PT: *Pan troglodytes*; GG: *Gorilla gorilla*; OG: *Otolemur garnettii*; BP: *Balaenoptera physalus*; CD: *Camelus dromedarius*; SS: *Sus scrofa*; CaLu: *Canis lupus*; MM: *Mus musculus*; RN: *Rattus norvegicus*; CG: *Cricetulus griseus*; ChLa: *Chinchilla lanigera*. The underlined sequence corresponds to that of exon 7. The sequences of the peptides that used to generate polyclonal antibodies are highlighted (Pept 1 and Pept 2). (D) RT‐PCR analyses of the expression of the different VEGF (black stars) and VEGF_XXX/NF_ isoforms (NF, red stars) in normal tissues (T, TIME endothelial Biocells, capital, kidney, Li, liver, Lu, lung). (E) RT‐PCR analyses of the expression of the different VEGF (black stars) and VEGF_XXX/NF_ isoforms (NF, red stars) in RCC cells (A, ACHN, 786, 786‐O). Red stars/red numbers show VEGF_XXX/NF_ isoforms; black stars/red numbers show VEGF_XXX_ isoforms. Results are presented as the mean ± SEM. Experiments were performed with at least three biological duplicates (n = 3) for each group in triplicate.

**Fig. S3.** Expression of VEGF_XXX/NF_ in cancer cells. Assessment of VEGF and VEGF_XXX/NF_ expression in breast cancer (MDA‐MB‐231), medulloblastoma (DAOY) and pancreatic ductal adenocarcinoma (MiaPaca‐2) cells by RT‐qPCR. ***P < 0.001 vs. VEGF in TIME, ### *P* < 0.001 vs. VEGF_XXX/NF_ in TIME. Results are presented as the mean ± SEM. Experiments were performed with at least three biological duplicates (n = 3) for each group in triplicate.

**Fig. S4.** Characterization of rabbit anti‐VEGF_XXX/NF_ antibodies. (A) Epitopes of anti‐VEGF_XXX/NF_ antibodies #1 and #2. (B) Two crude sera targeting epitope 1 (#1) and two crude sera targeting epitope 2 (#2) were analysed. Samples: 1) Empty vector (EV), 2) pcDNA3.1‐VEGF_222/NF_ were loaded onto an acrylamide gel and immunoblotted with four different antibodies and rabbit preimmune sera. Specific bands are highlighted by red asterisk. (C) Specificity of anti‐VEGF_XXX/NF_ antibodies. 5 ng (rVA) or 20 ng (rVA’) recombinant VEGF_165_ or 5 ng (rVB) or 20 ng (rVB’) recombinant VEGF_165b_ or conditioned medium from HEK293 cells transfected with empty vector (EV), a vector, encoding VEGF_165_ (pL6VA), or two independent vectors encoding VEGF_222/NF_ (pCNF) or (pL6NF) were loaded onto an acrylamide gel, and immunoblotting with the anti‐VEGF_XXX/NF_ #2.2 antibody was performed. (D) Cell lysates or conditioned media from HEK293 cells expressing EV, pL6VA, pCNF, pL6NF were loaded onto an acrylamide gel, and immunoblotting was performed using the anti‐VEGF_XXX/NF_ #2.2 antibody or anti‐HSP90 as loading control. Red asterisks indicate VEGF_222/NF_. Results are presented as representative images of three independent experiments.

**Fig. S5.** Affinity of VEGF_165_, VEGFC and VEGF_222/NF_ for the different VEGFR and VEGF coreceptors. (A) Association and dissociation sensogram/NFs obtained. (B) Kinetic values for the 15 measured interactions. *: Dissociation is too slow. The value measured by the instrument is not precise. Calculated KD value could be under or over estimated. Binding signal too low: a binding is detected but the binding signal is too low to estimate the kinetic values of the interaction.

**Fig. S6.** VEGF_222/NF_ induces endothelial cell activation and permeability. (A) *In vitro* permeability assay. A monolayer of serum‐depleted TIME cells on 4‐μm pore culture inserts was treated with VEGF_222/NF_ (100 ng/mL) in the presence of axitinib (1 μm) for 30 min. Streptavidin‐HRP was then added to the transwell for 10 min, and TMB substrate was added to the lower compartment to determine permeability. (B) Axitinib inhibits VEGF_222/NF_‐dependent activation of VEGFR2 and downstream ERK signalling pathway (representative blot of three independent experiments). *P* values are given. ** *P* < 0.01 (comparison of control vs VEGF_222/NF_ treated); # *P* < 0.01 (comparison VEGF_222/NF_ treated in the absence or presence of axitinib) (two‐way ANOVA). Results are presented as the mean ± SEM. Experiments were performed with at least three biological duplicates (n = 3) for each group in triplicate.

**Fig. S7.** VEGF_222/NF_ promotes proliferation and survival of RCC cells through NRP1 and NRP2. (A) RT‐qPCR analysis of VEGF_XXX/NF_ and VEGF expression in ACHN‐overexpressing VEGF_222/NF_ cells. ACHN cells were transduced with pLenti6.3 expressing full‐length VEGF_222/NF_ cDNA, and VEGF_XXX/NF_ mRNA expression was examined. Expression of VEGF_222/NF_ and VEGF_121_ is represented as percent of total VEGF and was normalized to the mean VEGF expression measured in ACHN‐LacZ cell defined as 100%. *** *P* < 0.001 vs LacZ. (B) ELISA of VEGF_XXX/NF_ and VEGF in the supernatant of ACHN‐overexpressing VEGF_222/NF_ cells. *** *P* < 0.001 vs LacZ. (C) Proliferation of ACHN‐overexpressing VEGF_222/NF_ cells. Cells were counted for 7 days. *** *P* < 0.001 vs LacZ. (D–E) RT‐qPCR analysis of VEGF_222/NF_ expression in ACHN (D) and 786‐O (E) cells transduced with pLKO.1 and sh expressing VEGF_222/NF_. * *P* < 0.05, ** *P* < 0.01, *** *P* < 0.001 vs scramble (Scr, two‐way ANOVA). (F) Clonogenic assay assessed with ACHN‐ (top) and in 786‐O‐ (bottom) ‐VEGF_XXX/NF_ downregulated cells 7 days after transduction. (G) mRNA expression of NRP1 and NRP2 in ACHN cells transfected with shScramble, shNRP1 or shNRP2. (H–I) Cell proliferation assay of ACHN‐VEGF_222/NF_ cells transfected with shNRP1 (H) or shNRP2 (I). ** *P* < 0.01, *** *P* < 0.001 vs shScramble. D0: day 0 (two‐way ANOVA). Results are presented as the mean ± SEM. Experiments were performed with at least three biological duplicates (n = 3) for each group in triplicate.

**Fig. S8.** VEGF_222/NF_ stimulates proliferation of human dermal lymphatic endothelial cells (HDLECs) and induces phosphorylation of VEGFR3. (A) HDLECs cells (25.000) were seeded in 6‐well plates in endothelial cell growth medium (Promocell) containing 0.5% FBS. Twenty‐four hours later, cells were treated with VEGF_165_ (100 ng/mL), VEGF_222/NF_ (100 ng/mL) or VEGFC (Sigma Aldrich, SRP3184) (100 ng/mL) (day 0) and were counted after 0, 24, 48 and 72 h. Results were expressed as fold increase with day 0 as reference. * *P* < 0.05, ** *P* < 0.01, *** *P* < 0.001 vs PBS, # *P* < 0.05, ## *P* < 0.01 vs VEGF_165_, § *P* < 0.05, §§ *P* < 0.01 vs VEGF_222/NF_ (two‐way ANOVA). (B) ELISA of p‐VEGFR3 activation. Phospho‐VEGFR3 levels were measured by ELISA (Human phospho‐VEGFR3 DuoSet IC ELISA, R&D systems, DYC2724) after starved HDLECs were treated with VEGF_165_, VEGF_222/NF_ or VEGFC (100 ng/mL) for 15 min. Results are expressed as pg phospho‐VEGFR3/μg proteins. ** *P* < 0.01 vs VEGF_222/NF_ (two‐way ANOVA). ND: Not detectable. Results are presented as the mean ± SEM. Experiments were performed with at least three biological duplicates (n = 3) for each group in triplicate.

**Fig. S9.** Anti‐VEGF_XXX/NF_ antibodies specifically recognize VEGF_222/NF_. (A) Immunoblotting. Recombinant KLH, VEGF_165_ or VEGF_222/NF_ (100 ng) were loaded onto acrylamide gels. Proteins were identified using the mouse anti‐VEGF_XXX/NF_ (1/2000). (B) ELISA. KLH and VEGF_222/NF_ (100 ng/well) were immobilized overnight on 96‐well plates and then incubated with the mouse anti‐VEGF_XXX/NF_ (1/2000). Detection was performed with TMB. Results are given as optical density values (OD). ** *P* < 0.01 vs KLH (two‐way ANOVA). Results are presented as the mean ± SEM. Experiments were performed with at least three biological duplicates (n = 3) for each group in triplicate.

**Fig. S10.** Characteristics of M1 ccRCC patients treated with sunitinib. T: size and/or extension of the original tumour (T1 to T4); N invasion of lymph node (0 no invasion; 1 invasion; x; lymph node cannot be assessed); M metastatic status (0 no metastasis; 1 presence of metastases). The Fuhrman grade is a histologic grading for RCC based on the microscopic morphology with haematoxylin and eosin staining.

**Fig. S11.** Characteristics of M1 ccRCC patients treated with bevacizumab plus interferon alpha or temsirolimus. T: size and /or extension of the original tumour (T1 to T4); N invasion of lymph node (0 no invasion; 1 invasion; x; lymph node cannot be assessed); M metastatic status (0 no metastasis; 1 presence of metastases).

**Fig. S12.** VEGF and VEGF_XXX/NF_ do not predict response to bevacizumab. (A–B) Levels of VEGF and VEGF_XXX/NF_ were assessed in plasma (just before bevacizumab + interferon or temsirolimus treatments) from 45 metastatic ccRCC patients. The third quartile was used as the cut‐off value for determining the patient group, that is, 4500 pg/mL and 3000 pg/mL for VEGF and VEGF_XXX/NF_, respectively. Correlation of plasma levels of VEGF (A–B) or VEGF_XXX/NF_ (C–D) with PFS and OS during first‐line treatment with bevacizumab + interferon or temsirolimus. The Kaplan–Meier method was used to generate survival curves, and Cox models were used to analyse the censored data. The statistical significance (P values) is given.

**Fig. S13.** Predictive value of VEGF and VEGF_XXX/NF_ co‐detection in M1 ccRCC patients. Plasma VEGF_XXX/NF_ levels were determined in metastatic ccRCC patients immediately prior to treatment with sunitinib. Levels of VEGF and VEGF_XXX/NF_ were assessed in plasma (just before sunitinib treatment) from 47 metastatic ccRCC patients (SUVEGIL and TORAVA cohorts). The third quartile was used as the cut‐off value (low or high) for determining the patient group, that is, 4500 pg/mL and 3000 pg/mL for VEGF and VEGF_XXX/NF_, respectively. The Kaplan–Meier method was used to generate survival curves, and Cox models were used to analyse the censored data. Statistical significance (*P* value) is indicated.

**Fig. S14.** VEGF_XXX/NF_ isoforms are expressed to varying degrees in 100% of ccRCC samples. Sixty independent tumour samples were analysed by qPCR. The extent of expression is expressed in arbitrary units. Mean expression is shown in the graph (first quartile 15 tumours, 0.014 < X < 0.059; second quartile 16 tumours, 0.06 < X < 0.17; third quartile 0.19 < X < 0.453; fourth quartile, 0.52 < X < 4.05).
