# Supplementary material for: A group of novel VEGF splice variants as alternative therapeutic targets in renal cell carcinoma
Source: Mol Oncol. 2023 Apr 18;17(7):1379–401. doi: 10.1002/1878-0261.13401 (PMC10323879; doi:10.1002/1878-0261.13401)
Supplement: Supplementary file 1 — Fig. S1. DNA and protein sequences of native and recombinant VEGF222/NF. (A) DNA sequence encoding the native VEGF222/NF protein and (B) the native VEGF222/NF protein. (C) DNA sequence encoding the optimized His‐tagged VEGF222/NF sequence and (D) the corresponding protein sequence produced by ProteoGenix. [file MOL2-17-1379-s002.pdf]

A

>VEGF$_{222/NF}$ – 747 bp native sequence

ATGAACTTTCTGCTGTCTTGGGTGCATTGGAGCCTTGCCTTGCTGCTCTACCTCCACCATGCCAAGTGGTCCCAGGCTGCAC
CCATGGCAGAAGGAGGAGGGCAGAATCATCACGAAGTGGTGAAGTTCATGGATGTCTATCAGCGCAGCTACTGCCATCCAAT
CGAGACCCTGGTGGACATCTTCCAGGAGTACCCTGATGAGATCGAGTACATCTTCAAGCCATCCTGTGTGCCCCTGATGCGA
TGCGGGGGCTGCTGCAATGACGAGGGCCTGGAGTGTGTGCCCACTGAGGAGTCCAACATCACCATGCAGATTATGCGGATCA
AACCTCACCAAGGCCAGCACATAGGAGAGATGAGCTTCCTACAGCACAACAAATGTGAATGCAGACCAAAGAAAGATAGAGC
AAGACAAGAAAATCCCTGTGGGCCTTGCTCAGAGCGGAGAAAGCATTTGTTTGTACAAGATCCGCAGACGTGTAAATGTTCC
TGCAAAAACACAGACTCGCGTTGCAAGGCGAGGCAGCTTGAGTTAAACGAACGTACTTGCAGCCTTTGTTTTCCATTTCCCT
CAGATGTGACAAGCCGAGGCGGTGAGCCGGGCAGGAGGAAGGAGCCTCCCTCAGGGTTTCGGGAACCAGATCTCTCACCAGG
AAAGACTGATACAGAACGATCGATACAGAAACCACGCTGCCGCCACCACACCATCACCATCGACAGAACAGTCCTTAATCCA
GAAACCTGA

B

>VEGF$_{222/NF}$ protein – 248 aa – 28,6 kDa

MNFLLSWVHWSLALLLYLHHAKWSQAAPMAEGGGQNHHEVVKFMDVYQRSYCHPIETLVDIFQEYPDEIEYIFKPSCVPLMR
CGGCCNDEGLECVPTEESNITMQIMRIKPHQGQHIGEMSFLQHNKCECRPKKDRARQENPCGPCSERRKHLFVQDPQTCKCS
CKNTDSRCKARQLELNERTCSLCFPFPSDVTSRGGEPGRRKEPPSGFREPDLSPGKTDTERSIQKPRCRHHTITIDRTVLNP
ET*

C

Proteogenix VEGF$_{222/NF}$ synthesis

>SlP-HIs-3C-VEGF_222/NF - 807 bp

ATGAACTTCTACAAGATCTTCGTCTTCGTCGCCCTGATCCTGGCCATCAGCATCGGTCAGAGCGAAGCTGGTAGCCACCACC
ACCACCATCACAGCGGTCTGGAAGTGCTGTTCCAGGGTCCTGCCCCTATGGCTGAGGGCGGTGGTCAGAACCACCACGAAGT
GGTCAAGTTCATGGACGTGTACCAGCGCAGCTACTGCCACCCTATCGAAACTCTGGTGGACATCTTCCAGGAGTACCCTGAC
GAGATCGAGTACATCTTCAAGCCTAGCTGCGTGCCTCTGATGCGTTGCGGTGGTTGCTGCAACGACGAGGGTCTGGAGTGCG
TGCCTACTGAAGAGAGCAACATCACTATGCAGATCATGCGCATCAAGCCTCACCAGGGTCAGCACATCGGTGAAATGAGCTT
CCTGCAGCACAACAAGTGCGAATGCCGCCCTAAGAAGGACCGCGCTCGCCAGGAAAACCCTTGCGGTCCTTGCTCCGAGCGT
CGCAAGCACCTGTTCGTGCAGGACCCCCAGACCTGCAAGTGCTCCTGCAAGAACACTGACTCCCGTTGCAAGGCCCGTCAGC
TGGAGCTGAACGAGCGCACCTGCAGCCTGTGCTTCCCTTTCCCCAGCGACGTCACTTCCCGTGGCGGCGAGCCTGGTCGCCG
TAAGGAACCCCCTAGCGGTTTCCGCGAACCCGACCTGTCCCCCGGTAAAACCGACACCGAGCGTAGCATCCAGAAGCCTCGC
TGCCGTCACCACACCATCACCATCGACCGCACCGTGCTGAACCCTGAAACCTAA

D

>SlP-HIs-3C-VEGF$_{222/NF}$ - 263 AAs- 29.92kDa

MNFYKIFVFVALILAISIGQSEAGSHHHHHHSGLEVLFQGPAPMAEGGGQNHHEVVKFMDVYQRSYCHPIETLVDIFQEYPD
EIEYIFKPSCVPLMRCGGCCNDEGLECVPTEESNITMQIMRIKPHQGQHIGEMSFLQHNKCECRPKKDRARQENPCGPCSER
RKHLFVQDPQTCKCSCKNTDSRCKARQLELNERTCSLCFPFPSDVTSRGGEPGRRKEPPSGFREPDLSPGKTDTERSIQKPR
CRHHTITIDRTVLNPET

Features: Signal peptide: [1:23]; His-tag with linker: [24:33]

Supplementary Figure 1: Montemagno *et al*
