# Supplementary figures and images for: A group of novel VEGF splice variants as alternative therapeutic targets in renal cell carcinoma
Source: Mol Oncol. 2023 Apr 18;17(7):1379–401. doi: 10.1002/1878-0261.13401 (PMC10323879; doi:10.1002/1878-0261.13401)

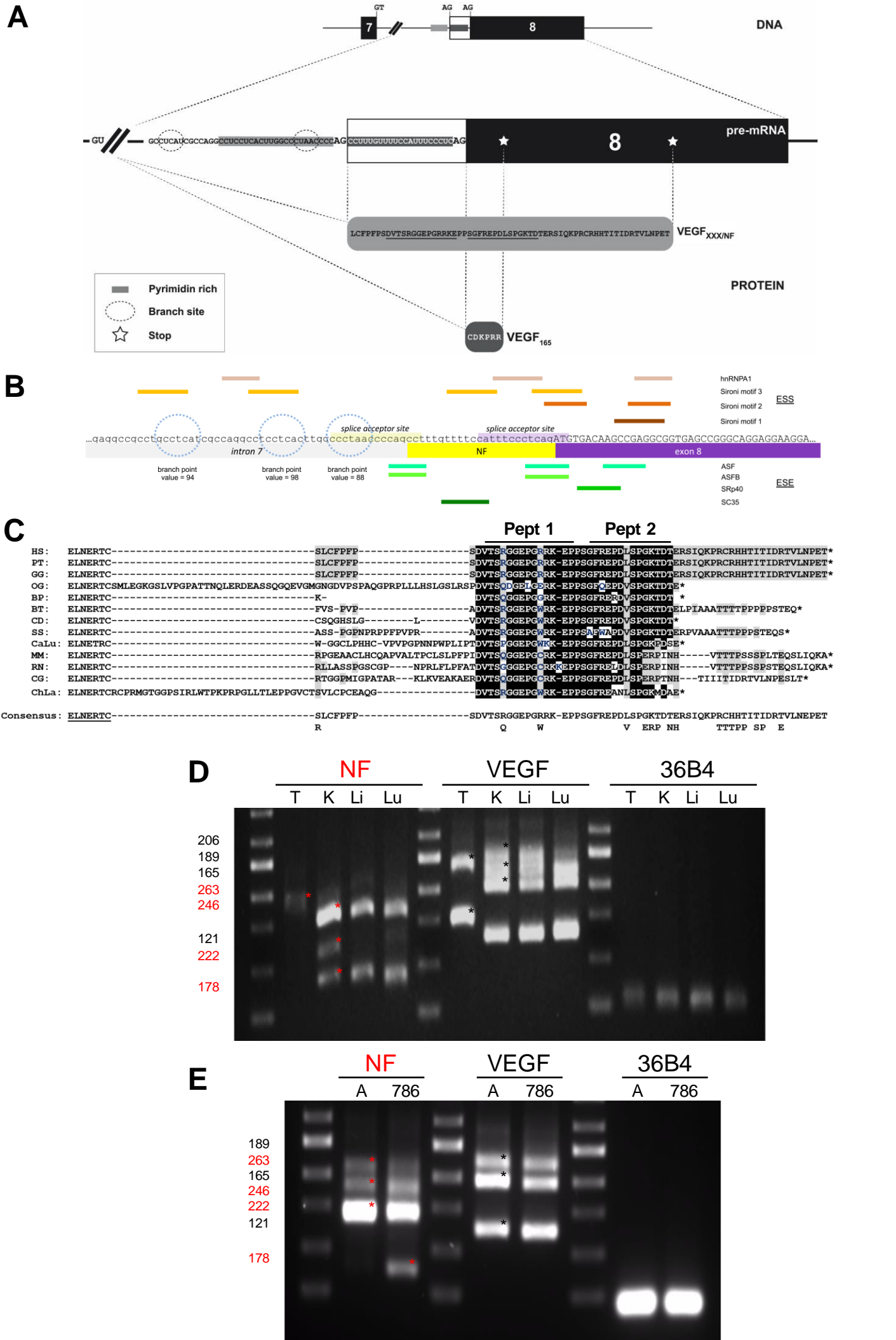

Supplementary Figure 2: Montemagno *et al*

Supplement: Supplementary file 2 — Fig. S2. Bioinformatic analysis of the last intron of the human VEGF gene and conservation of the protein from the resulting splicing event in different mammals. (A) Possible splicing events of VEGF pre‐mRNA and the resulting C‐terminal specific sequence of VEGFXXX/NF and VEGF165. (B) Bioinformatic analysis of the intron 7 exon 8 part with ESS and ESE motifs. (C) Conservation of the C‐terminal sequence of VEGFXXX/NF between species. HS: Homo sapiens; PT: Pan troglodytes; GG: Gorilla gorilla; OG: Otolemur garnettii; BP: Balaenoptera physalus; CD: Camelus dromedarius; SS: Sus scrofa; CaLu: Canis lupus; MM: Mus musculus; RN: Rattus norvegicus; CG: Cricetulus griseus; ChLa: Chinchilla lanigera. The underlined sequence corresponds to that of exon 7. The sequences of the peptides that used to generate polyclonal antibodies are highlighted (Pept 1 and Pept 2). (D) RT‐PCR analyses of the expression of the different VEGF (black stars) and VEGFXXX/NF isoforms (NF, red stars) in normal tissues (T, TIME endothelial Biocells, capital, kidney, Li, liver, Lu, lung). (E) RT‐PCR analyses of the expression of the different VEGF (black stars) and VEGFXXX/NF isoforms (NF, red stars) in RCC cells (A, ACHN, 786, 786‐O). Red stars/red numbers show VEGFXXX/NF isoforms; black stars/red numbers show VEGFXXX isoforms. Results are presented as the mean ± SEM. Experiments were performed with at least three biological duplicates (n = 3) for each group in triplicate. [file MOL2-17-1379-s005.pdf]

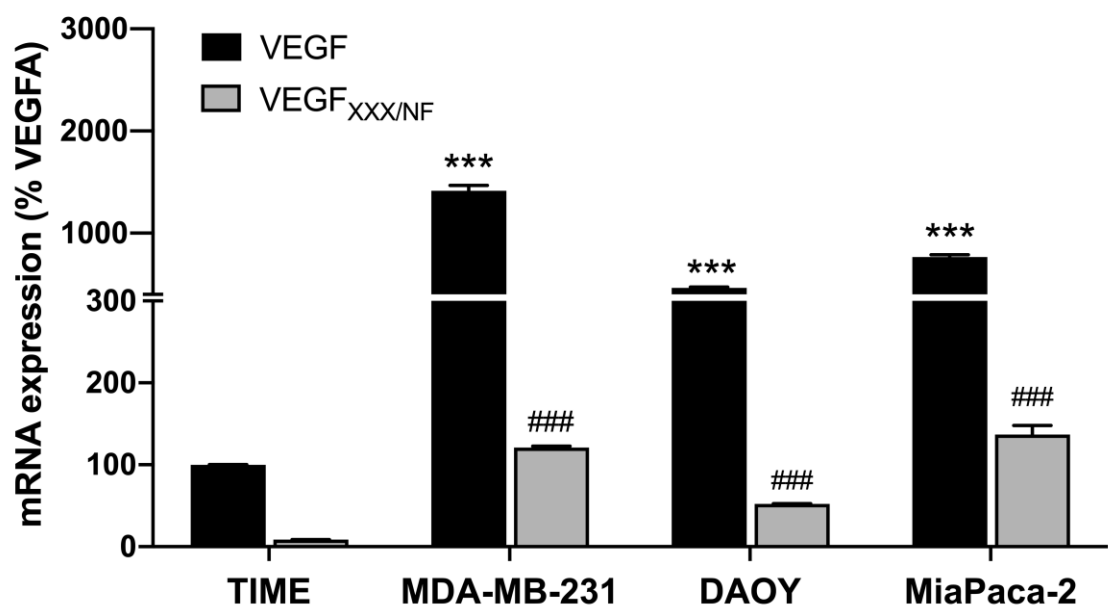

**Supplementary Figure 3: Montemagno *et al***

Supplement: Supplementary file 3 — Fig. S3. Expression of VEGFXXX/NF in cancer cells. Assessment of VEGF and VEGFXXX/NF expression in breast cancer (MDA‐MB‐231), medulloblastoma (DAOY) and pancreatic ductal adenocarcinoma (MiaPaca‐2) cells by RT‐qPCR. ***P < 0.001 vs. VEGF in TIME, ### P < 0.001 vs. VEGFXXX/NF in TIME. Results are presented as the mean ± SEM. Experiments were performed with at least three biological duplicates (n = 3) for each group in triplicate. [file MOL2-17-1379-s014.pdf]

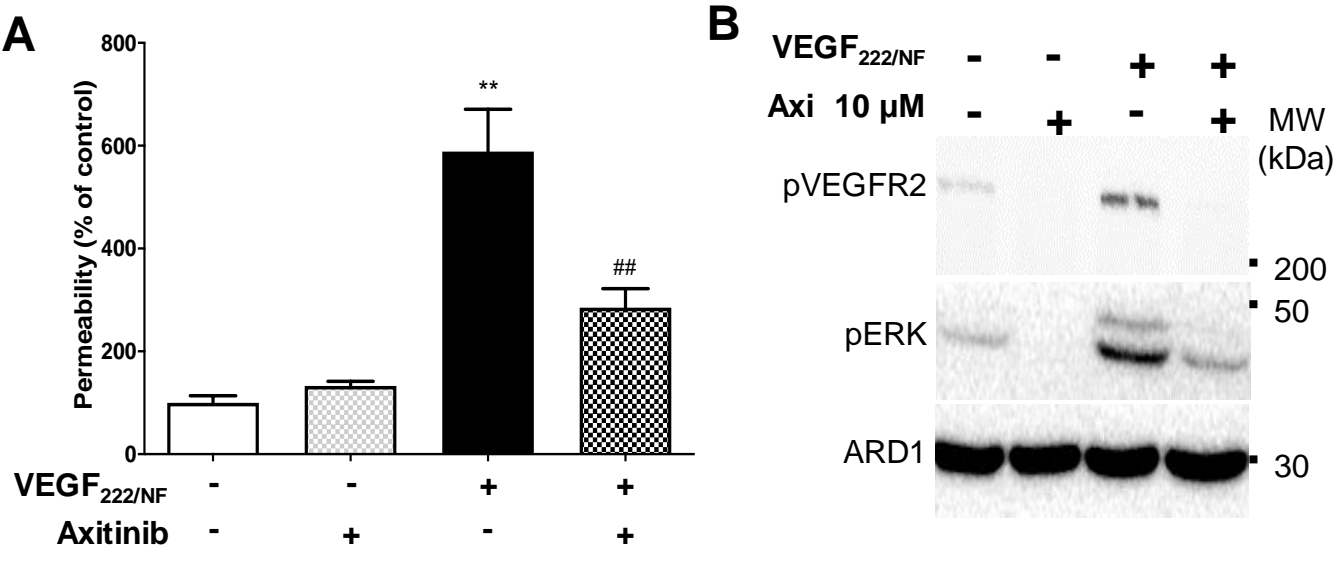

Supplementary Figure 6: Montemagno *et al*

Supplement: Supplementary file 6 — Fig. S6. VEGF222/NF induces endothelial cell activation and permeability. (A) In vitro permeability assay. A monolayer of serum‐depleted TIME cells on 4‐μm pore culture inserts was treated with VEGF222/NF (100 ng/mL) in the presence of axitinib (1 μm) for 30 min. Streptavidin‐HRP was then added to the transwell for 10 min, and TMB substrate was added to the lower compartment to determine permeability. (B) Axitinib inhibits VEGF222/NF‐dependent activation of VEGFR2 and downstream ERK signalling pathway (representative blot of three independent experiments). P values are given. ** P < 0.01 (comparison of control vs VEGF222/NF treated); # P < 0.01 (comparison VEGF222/NF treated in the absence or presence of axitinib) (two‐way ANOVA). Results are presented as the mean ± SEM. Experiments were performed with at least three biological duplicates (n = 3) for each group in triplicate. [file MOL2-17-1379-s007.pdf]

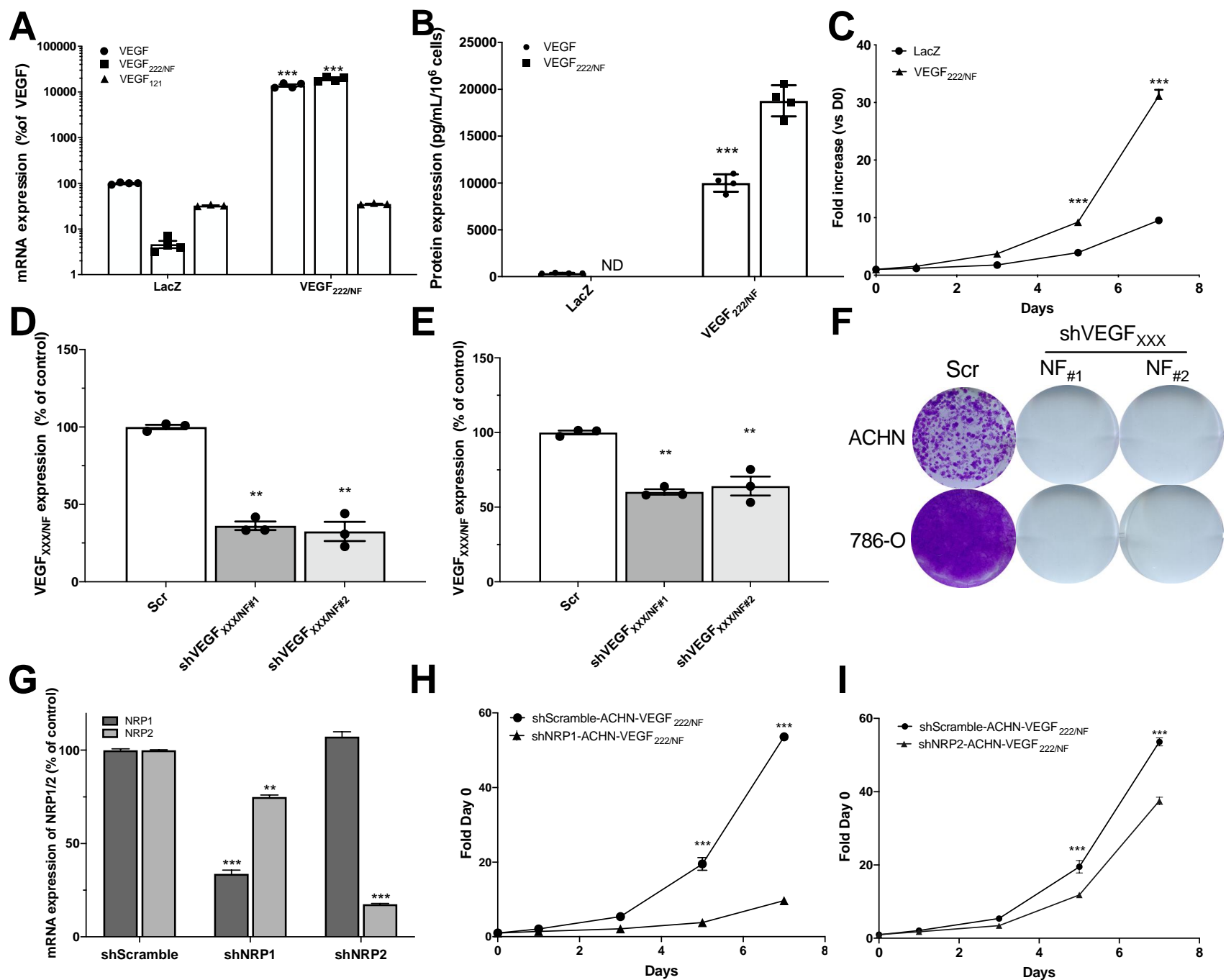

**Supplementary Figure 7: Montemagno *et al***

Supplement: Supplementary file 7 — Fig. S7. VEGF222/NF promotes proliferation and survival of RCC cells through NRP1 and NRP2. (A) RT‐qPCR analysis of VEGFXXX/NF and VEGF expression in ACHN‐overexpressing VEGF222/NF cells. ACHN cells were transduced with pLenti6.3 expressing full‐length VEGF222/NF cDNA, and VEGFXXX/NF mRNA expression was examined. Expression of VEGF222/NF and VEGF121 is represented as percent of total VEGF and was normalized to the mean VEGF expression measured in ACHN‐LacZ cell defined as 100%. *** P < 0.001 vs LacZ. (B) ELISA of VEGFXXX/NF and VEGF in the supernatant of ACHN‐overexpressing VEGF222/NF cells. *** P < 0.001 vs LacZ. (C) Proliferation of ACHN‐overexpressing VEGF222/NF cells. Cells were counted for 7 days. *** P < 0.001 vs LacZ. (D–E) RT‐qPCR analysis of VEGF222/NF expression in ACHN (D) and 786‐O (E) cells transduced with pLKO.1 and sh expressing VEGF222/NF. * P < 0.05, ** P < 0.01, *** P < 0.001 vs scramble (Scr, two‐way ANOVA). (F) Clonogenic assay assessed with ACHN‐ (top) and in 786‐O‐ (bottom) ‐VEGFXXX/NF downregulated cells 7 days after transduction. (G) mRNA expression of NRP1 and NRP2 in ACHN cells transfected with shScramble, shNRP1 or shNRP2. (H–I) Cell proliferation assay of ACHN‐VEGF222/NF cells transfected with shNRP1 (H) or shNRP2 (I). ** P < 0.01, *** P < 0.001 vs shScramble. D0: day 0 (two‐way ANOVA). Results are presented as the mean ± SEM. Experiments were performed with at least three biological duplicates (n = 3) for each group in triplicate. [file MOL2-17-1379-s011.pdf]

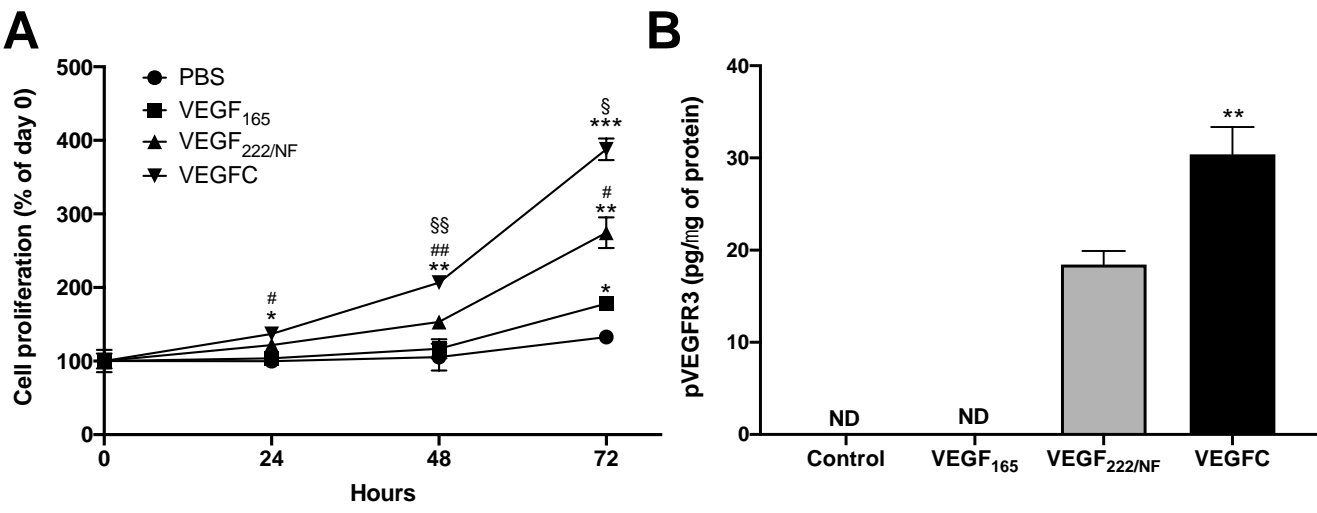

Supplementary Figure 8: Montemagno *et al*

Supplement: Supplementary file 8 — Fig. S8. VEGF222/NF stimulates proliferation of human dermal lymphatic endothelial cells (HDLECs) and induces phosphorylation of VEGFR3. (A) HDLECs cells (25.000) were seeded in 6‐well plates in endothelial cell growth medium (Promocell) containing 0.5% FBS. Twenty‐four hours later, cells were treated with VEGF165 (100 ng/mL), VEGF222/NF (100 ng/mL) or VEGFC (Sigma Aldrich, SRP3184) (100 ng/mL) (day 0) and were counted after 0, 24, 48 and 72 h. Results were expressed as fold increase with day 0 as reference. * P < 0.05, ** P < 0.01, *** P < 0.001 vs PBS, # P < 0.05, ## P < 0.01 vs VEGF165, § P < 0.05, §§ P < 0.01 vs VEGF222/NF (two‐way ANOVA). (B) ELISA of p‐VEGFR3 activation. Phospho‐VEGFR3 levels were measured by ELISA (Human phospho‐VEGFR3 DuoSet IC ELISA, R&D systems, DYC2724) after starved HDLECs were treated with VEGF165, VEGF222/NF or VEGFC (100 ng/mL) for 15 min. Results are expressed as pg phospho‐VEGFR3/μg proteins. ** P < 0.01 vs VEGF222/NF (two‐way ANOVA). ND: Not detectable. Results are presented as the mean ± SEM. Experiments were performed with at least three biological duplicates (n = 3) for each group in triplicate. [file MOL2-17-1379-s013.pdf]

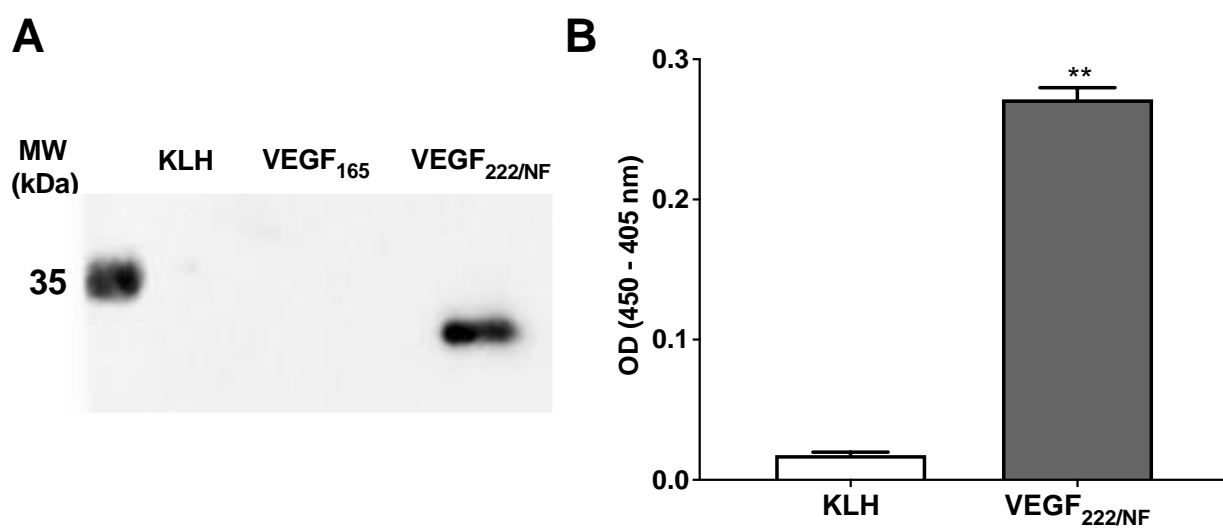

**Supplementary Figure 9: Montemagno *et al***

Supplement: Supplementary file 9 — Fig. S9. Anti‐VEGFXXX/NF antibodies specifically recognize VEGF222/NF. (A) Immunoblotting. Recombinant KLH, VEGF165 or VEGF222/NF (100 ng) were loaded onto acrylamide gels. Proteins were identified using the mouse anti‐VEGFXXX/NF (1/2000). (B) ELISA. KLH and VEGF222/NF (100 ng/well) were immobilized overnight on 96‐well plates and then incubated with the mouse anti‐VEGFXXX/NF (1/2000). Detection was performed with TMB. Results are given as optical density values (OD). ** P < 0.01 vs KLH (two‐way ANOVA). Results are presented as the mean ± SEM. Experiments were performed with at least three biological duplicates (n = 3) for each group in triplicate. [file MOL2-17-1379-s003.pdf]

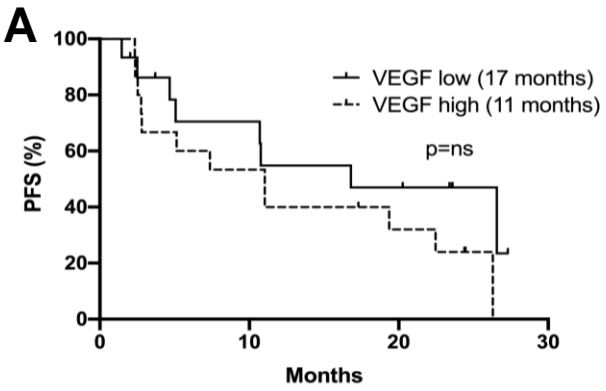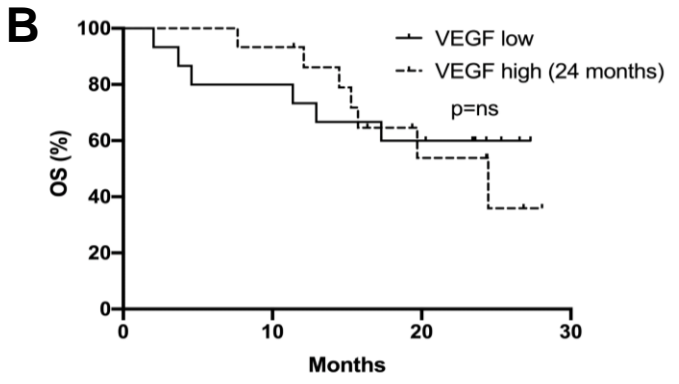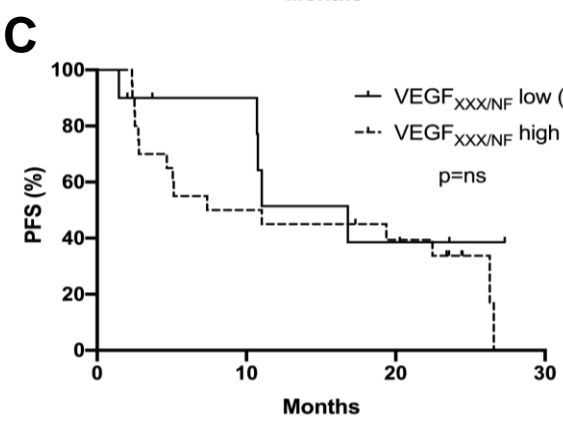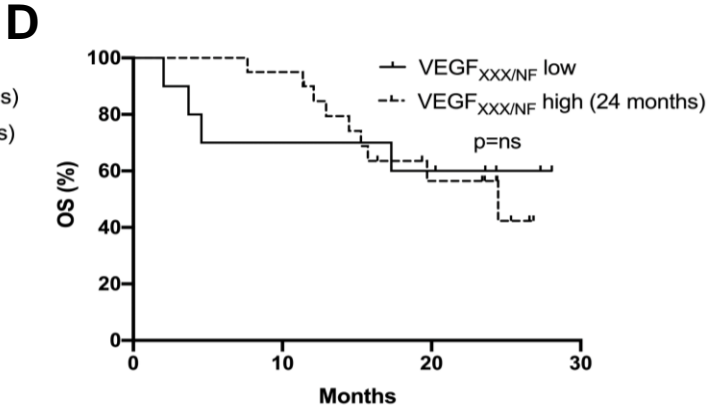

**Supplementary Figure 12: Montemagno *et al***

Supplement: Supplementary file 12 — Fig. S12. VEGF and VEGFXXX/NF do not predict response to bevacizumab. (A–B) Levels of VEGF and VEGFXXX/NF were assessed in plasma (just before bevacizumab + interferon or temsirolimus treatments) from 45 metastatic ccRCC patients. The third quartile was used as the cut‐off value for determining the patient group, that is, 4500 pg/mL and 3000 pg/mL for VEGF and VEGFXXX/NF, respectively. Correlation of plasma levels of VEGF (A–B) or VEGFXXX/NF (C–D) with PFS and OS during first‐line treatment with bevacizumab + interferon or temsirolimus. The Kaplan–Meier method was used to generate survival curves, and Cox models were used to analyse the censored data. The statistical significance (P values) is given. [file MOL2-17-1379-s004.pdf]

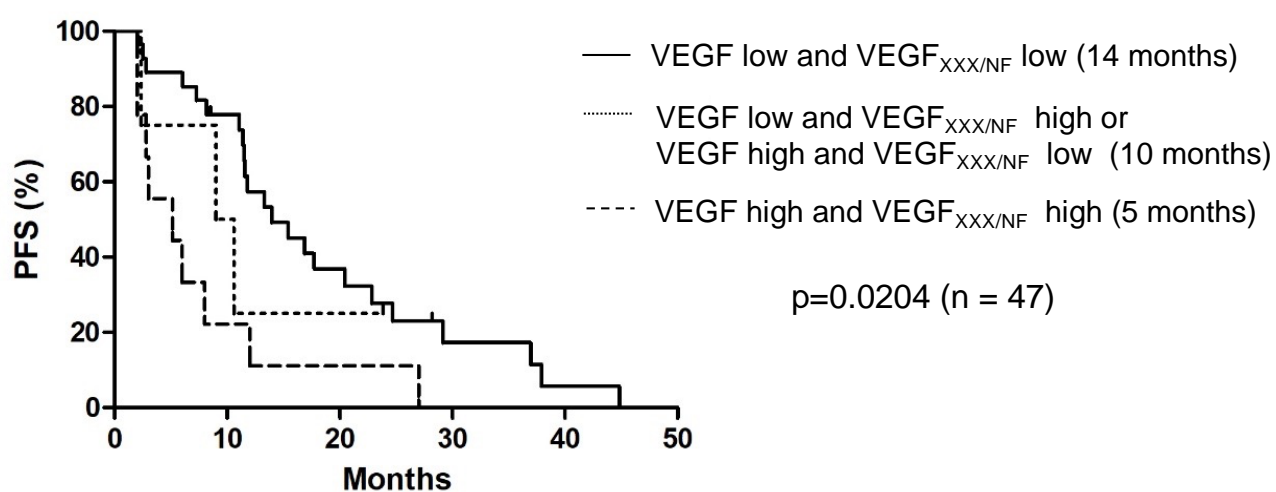

**Supplementary Figure 13: Montemagno *et al***

Supplement: Supplementary file 13 — Fig. S13. Predictive value of VEGF and VEGFXXX/NF co‐detection in M1 ccRCC patients. Plasma VEGFXXX/NF levels were determined in metastatic ccRCC patients immediately prior to treatment with sunitinib. Levels of VEGF and VEGFXXX/NF were assessed in plasma (just before sunitinib treatment) from 47 metastatic ccRCC patients (SUVEGIL and TORAVA cohorts). The third quartile was used as the cut‐off value (low or high) for determining the patient group, that is, 4500 pg/mL and 3000 pg/mL for VEGF and VEGFXXX/NF, respectively. The Kaplan–Meier method was used to generate survival curves, and Cox models were used to analyse the censored data. Statistical significance (P value) is indicated. [file MOL2-17-1379-s012.pdf]

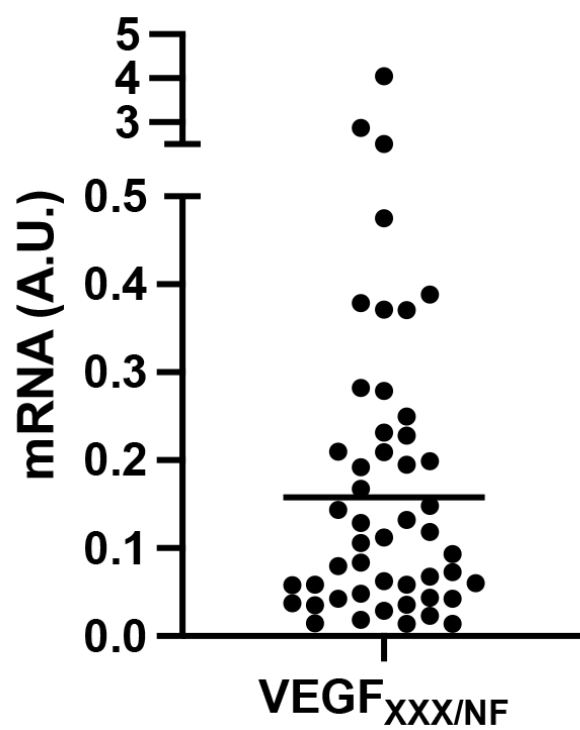

Supplementary Figure 14: Montemagno *et al*

Supplement: Supplementary file 14 — Fig. S14. VEGFXXX/NF isoforms are expressed to varying degrees in 100% of ccRCC samples. Sixty independent tumour samples were analysed by qPCR. The extent of expression is expressed in arbitrary units. Mean expression is shown in the graph (first quartile 15 tumours, 0.014 < X < 0.059; second quartile 16 tumours, 0.06 < X < 0.17; third quartile 0.19 < X < 0.453; fourth quartile, 0.52 < X < 4.05). [file MOL2-17-1379-s001.pdf]
