# Supplementary material for: A group of novel VEGF splice variants as alternative therapeutic targets in renal cell carcinoma
Source: Mol Oncol. 2023 Apr 18;17(7):1379–401. doi: 10.1002/1878-0261.13401 (PMC10323879; doi:10.1002/1878-0261.13401)
Supplement: Supplementary file 4 — Fig. S4. Characterization of rabbit anti‐VEGFXXX/NF antibodies. (A) Epitopes of anti‐VEGFXXX/NF antibodies #1 and #2. (B) Two crude sera targeting epitope 1 (#1) and two crude sera targeting epitope 2 (#2) were analysed. Samples: 1) Empty vector (EV), 2) pcDNA3.1‐VEGF222/NF were loaded onto an acrylamide gel and immunoblotted with four different antibodies and rabbit preimmune sera. Specific bands are highlighted by red asterisk. (C) Specificity of anti‐VEGFXXX/NF antibodies. 5 ng (rVA) or 20 ng (rVA’) recombinant VEGF165 or 5 ng (rVB) or 20 ng (rVB’) recombinant VEGF165b or conditioned medium from HEK293 cells transfected with empty vector (EV), a vector, encoding VEGF165 (pL6VA), or two independent vectors encoding VEGF222/NF (pCNF) or (pL6NF) were loaded onto an acrylamide gel, and immunoblotting with the anti‐VEGFXXX/NF #2.2 antibody was performed. (D) Cell lysates or conditioned media from HEK293 cells expressing EV, pL6VA, pCNF, pL6NF were loaded onto an acrylamide gel, and immunoblotting was performed using the anti‐VEGFXXX/NF #2.2 antibody or anti‐HSP90 as loading control. Red asterisks indicate VEGF222/NF. Results are presented as representative images of three independent experiments. [file MOL2-17-1379-s010.pdf]

A

VEGFNF Epitope #1	VEGFNF Epitope #2
DVTSRGGEPGRRKE	SGFREPDLSPGKTD

B

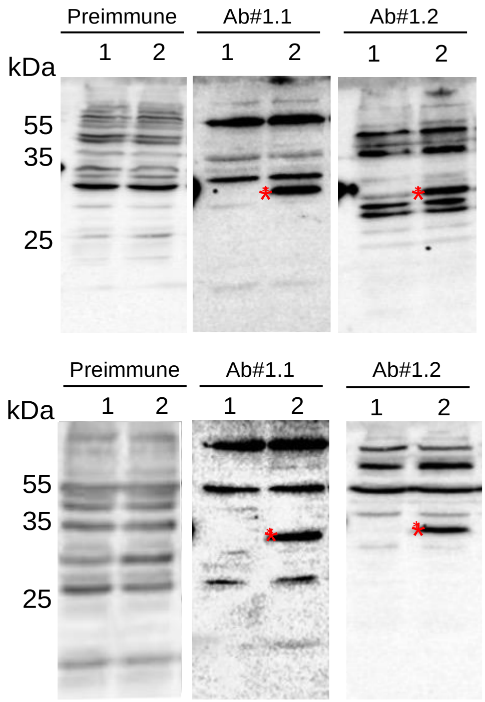

C

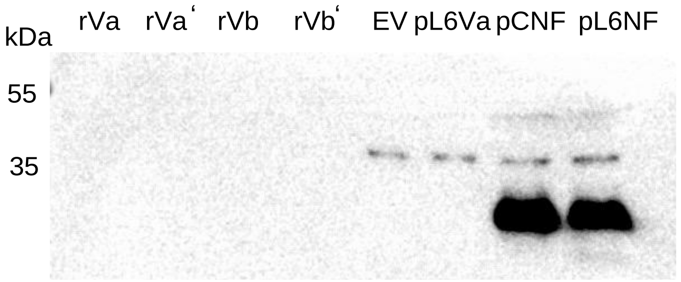

D

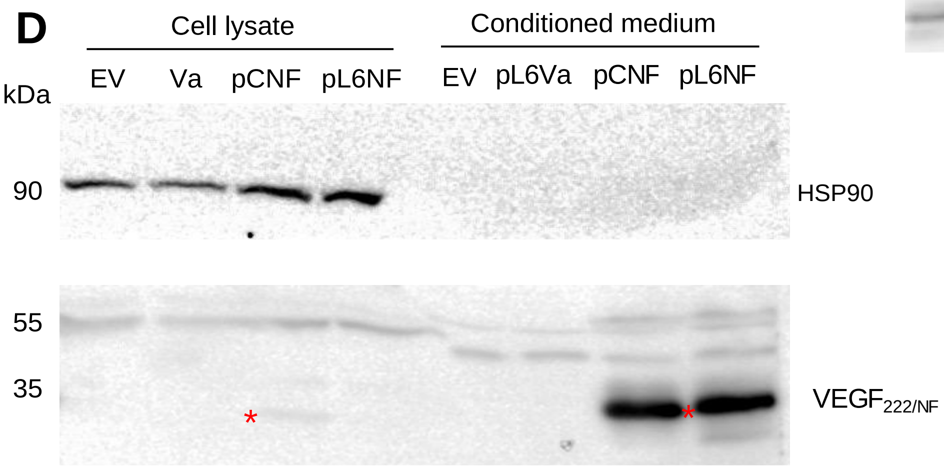

Supplementary Figure 4: Montemagno *et al*
