# Supplementary material for: A group of novel VEGF splice variants as alternative therapeutic targets in renal cell carcinoma
Source: Mol Oncol. 2023 Apr 18;17(7):1379–401. doi: 10.1002/1878-0261.13401 (PMC10323879; doi:10.1002/1878-0261.13401)
Supplement: Supplementary file 5 — Fig. S5. Affinity of VEGF165, VEGFC and VEGF222/NF for the different VEGFR and VEGF coreceptors. (A) Association and dissociation sensogram/NFs obtained. (B) Kinetic values for the 15 measured interactions. *: Dissociation is too slow. The value measured by the instrument is not precise. Calculated KD value could be under or over estimated. Binding signal too low: a binding is detected but the binding signal is too low to estimate the kinetic values of the interaction. [file MOL2-17-1379-s008.pdf]

A

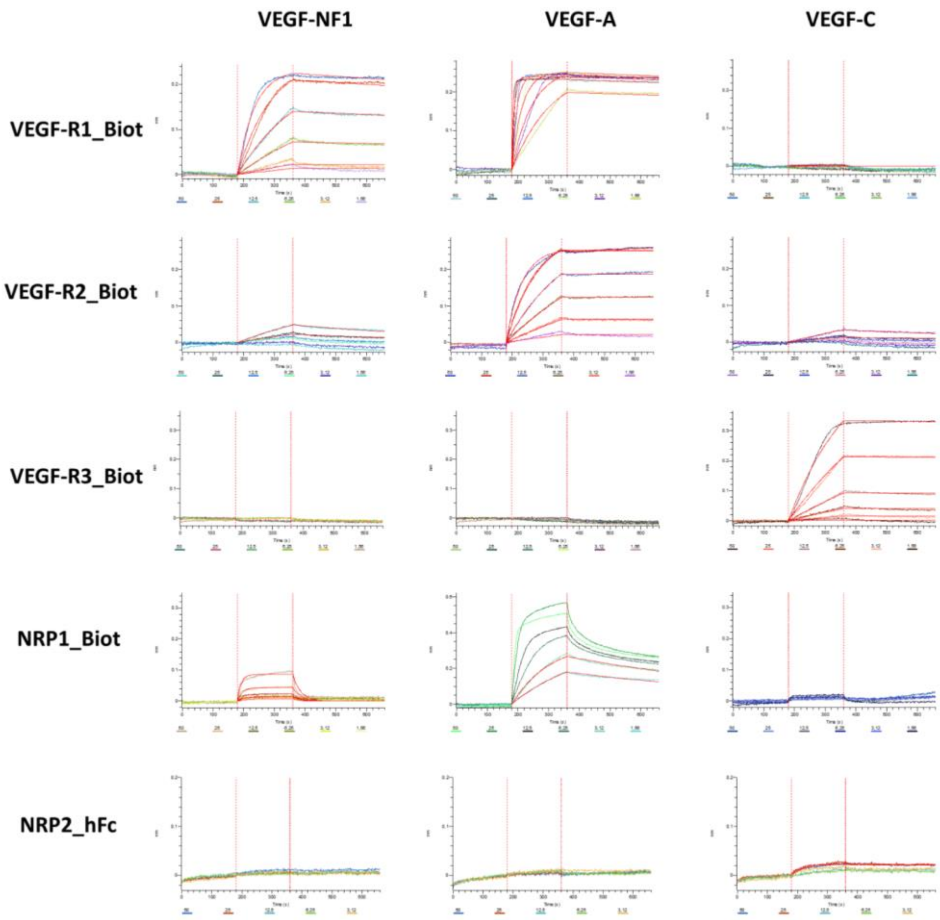

B

Immobilized ligand	Soluble anlayte	K_D (M)	ka (1/Ms)	kdis (1/s)
VEGF-R1_Biot	VEGF-NF1	5,48E-10	3,65E+05	2,00E-04
VEGF-R1_Biot	VEGF-A	2,46E-11	4,86E+06	1,19E-04
VEGF-R1_Biot	VEGC-C		No Binding	
VEGF-R2_Biot	VEGF-NF1		Binding signal too low	
VEGF-R2_Biot	VEGF-A	1,09E-12*	3,95E+05	4,29E-07*
VEGF-R2_Biot	VEGC-C		Binding signal too low	
VEGF-R3_Biot	VEGF-NF1		No Binding	
VEGF-R3_Biot	VEGF-A		No Binding	
VEGF-R3_Biot	VEGC-C	4,44E-10	9,65E+04	4,28E-05
NRP1_Biot	VEGF-NF1	4,12E-08	9,63E+05	3,97E-02
NRP1_Biot	VEGF-A	5,59E-10	2,21E+06	1,24E-03
NRP1_Biot	VEGC-C		Binding signal too low	
NRP2_Fc	VEGF-NF1		No Binding	
NRP2_Fc	VEGF-A		No Binding	
NRP2_Fc	VEGC-C		Binding signal too low	

Supplementary Figure 5: Montemagno *et al*
