# Supplementary material for: A group of novel VEGF splice variants as alternative therapeutic targets in renal cell carcinoma
Source: Mol Oncol. 2023 Apr 18;17(7):1379–401. doi: 10.1002/1878-0261.13401 (PMC10323879; doi:10.1002/1878-0261.13401)
Supplement: Supplementary file 10 — Fig. S10. Characteristics of M1 ccRCC patients treated with sunitinib. T: size and/or extension of the original tumour (T1 to T4); N invasion of lymph node (0 no invasion; 1 invasion; x; lymph node cannot be assessed); M metastatic status (0 no metastasis; 1 presence of metastases). The Fuhrman grade is a histologic grading for RCC based on the microscopic morphology with haematoxylin and eosin staining. [file MOL2-17-1379-s006.pdf]

	Total	Low VEGF$_{xxx/NF}$	High VEGF$_{xxx/NF}$	p value
**Number**	47	35	12	
**Age**	61.6 (30-81.3)	63.6 (30-81.3)	57 (43-70)	0.0271
**Sex**				ns
Female	11 (23.4%)	9 (25.7%)	2 (16.7%)	
Male	36 (76.6%)	26 (74.3%)	10 (83.3%)	
**pT**				0.042
1/2	15 (31.9%)	14 (40%)	1 (8.3%)	
3/4	32 (68.1%)	21 (60%)	11 (91.7%)	
**pN**				ns
0	26 (53.2%)	20 (57.2%)	6 (50%)	
≥ 1	6 (12.8%)	4 (11.4%)	2 (16.7%)	
x	15 (31.9%)	11 (31.4%)	4 (33.3%)	
**pM**				ns
0	26 (55.3%)	21 (60%)	5 (41.7%)	
1	21 (44.7%)	14 (40%)	7 (58.3%)	
**Fuhrman grade**				ns
1/2	14 (29.8%)	13 (37.2%)	1 (8.3%)	
3/4	33 (70.2%)	22 (62.8%)	11 (91.7%)	
**PFS (months) / progression %**	12<br>82%	15<br>76%	5<br>83%	0.0179
**OS (months) / Death %**	33<br>75%	37<br>68%	13<br>83%	0.0243

Supplementary Figure 10: Montemagno *et al*
