# Supplementary material for: A group of novel VEGF splice variants as alternative therapeutic targets in renal cell carcinoma
Source: Mol Oncol. 2023 Apr 18;17(7):1379–401. doi: 10.1002/1878-0261.13401 (PMC10323879; doi:10.1002/1878-0261.13401)
Supplement: Supplementary file 11 — Fig. S11. Characteristics of M1 ccRCC patients treated with bevacizumab plus interferon alpha or temsirolimus. T: size and /or extension of the original tumour (T1 to T4); N invasion of lymph node (0 no invasion; 1 invasion; x; lymph node cannot be assessed); M metastatic status (0 no metastasis; 1 presence of metastases). [file MOL2-17-1379-s009.pdf]

	Total	Low $VEGF_{NF}$	High $VEGF_{NF}$	p value
**Number**	30	10	20	
**Age**	57.7 (40-77.8)	58.8 (40-75.2)	57.3 (44.1-77.8)	0.99
**Sex**				ns
**Female**	8 (26.7%)	3 (30%)	5 (25%)	
**Male**	22 (73.3%)	7 (70%)	15 (75%)	
**pT**				ns
**1/2**	14 (46.7%)	4 (40%)	10 (50%)	
**3/4**	16 (53.3%)	6 (60%)	10 (50%)	
**pN**				ns
**x**	30 (100%)	10 (100%)	20 (100%)	
**pM**				0.03
**0**	19 (63.3%)	9 (90%)	10 (50%)	
**1**	11 (36.7%)	1 (10%)	10 (50%)	
**Fuhrman grade**				ns
**1/2**	11 (33.7%)	3 (30%)	8 (40%)	
**3/4**	19 (63.3%)	7 (70%)	12 (60%)	
**PFS (months) / progression %**	11<br>66.7%	16.8<br>50%	9.2<br>75%	0.402
**OS (months) / Death %**	24.5<br>43.3%	NR<br>40%	24.5<br>45%	0.93

Supplementary Figure 11: Montemagno *et al*
